# Interactive effects of sedimentary turbidity and elevated water temperature on the Pugnose Shiner (*Miniellus anogenus*), a threatened freshwater fish

**DOI:** 10.1093/conphys/coae053

**Published:** 2024-08-13

**Authors:** Liana Fortin-Hamel, Lauren J Chapman

**Affiliations:** Department of Biology, McGill University, 1205 avenue du Docteur-Penfield, Montreal, Quebec, Canada, H3A 1B1; Department of Biology, McGill University, 1205 avenue du Docteur-Penfield, Montreal, Quebec, Canada, H3A 1B1

**Keywords:** fish behaviour, fish physiology, fish swimming activity, hypoxia tolerance, imperilled fish, thermal tolerance

## Abstract

High turbidity and elevated water temperature are environmental stressors that can co-occur in freshwater ecosystems such as when deforestation increases solar radiation and sedimentary runoff. However, we have limited knowledge about their combined impacts on fish behaviour and physiology. We explored independent and interactive effects of sedimentary turbidity and temperature on the swimming activity and both thermal and hypoxia tolerance of the Pugnose Shiner (*Miniellus anogenus*, formerly *Notropis anogenus*), a small leuciscid fish listed as Threatened under Canada’s Species at Risk Act (SARA). Fish underwent a 15-week acclimation to two temperatures (16°C or 25°C) crossed with two turbidities (~0 NTU or 8.5 NTU). Swimming activity was measured during the first 8 weeks of acclimation. Fish in warm water were more active compared to those in cold water, but turbidity had no effect on activity. Behavioural response to hypoxia was measured after 12 weeks of acclimation, as the oxygen level at which fish used aquatic surface respiration (ASR). Fish in warm water engaged in ASR behaviour at higher oxygen thresholds, indicating less tolerance to hypoxia. Turbidity had no effect on ASR thresholds. Finally, thermal tolerance was measured as the critical thermal maximum (CT_max_) after 13–15 weeks of acclimation. Acclimation to warm water increased fish CT_max_ and T_ag_ (agitation temperature) but reduced the agitation window (°C difference between T_ag_ and CT_max_) and thermal safety margin (°C difference between the acclimation temperature and CT_max_). Furthermore, fish in warm, turbid water had a lower CT_max_ and smaller thermal safety margin than fish in warm, clear water, indicating an interaction between turbidity and temperature. This reduced thermal tolerance observed in Pugnose Shiner in warm, turbid water highlights the importance of quantifying independent and interactive effects of multiple stressors when evaluating habitat suitability and conservation strategies for imperilled species.

## Teaser

Threatened Pugnose Shiner (Miniellus anogenus) were acclimated to low and high water temperatures crossed with low and high turbidity levels over 15 weeks. We found that fish activity and sensitivity to hypoxia increased with temperature. Additionally, acclimation to both high temperature and high turbidity resulted in lower thermal tolerance.

## Introduction

Freshwaters are among the most disturbed ecosystems on our planet due to multiple anthropogenic threats such as land conversion, contaminant and sediment inflow, species introductions and overharvesting (Strayer and Dudgeon, 2010; Arthington *et al.*, 2016; Reid *et al.*, 2019). In addition, climate change is a growing threat to freshwaters that can lead to increased average water temperature, increased frequency of high temperature extremes and changes in precipitation regimes (Heino *et al.*, 2009; Knouft and Ficklin, 2017). Given the imperilled status of an estimated 30% of freshwater fishes (WWF, 2021) and the limited dispersal potential for many species inhabiting landlocked ecosystems (Woodward *et al.*, 2010), there is increasing interest in understanding the independent and interactive effects of multiple stressors on fitness-related traits (Ormerod *et al.*, 2010; Castañeda *et al.*, 2021). Two abiotic stressors that are increasingly co-occurring in freshwater systems and may have interactive effects on fishes are high turbidity (amount of suspended organic or sedimentary particles; Kale, 2016) and elevated water temperature. In this study, we explored independent and interactive effects of these two stressors on behavioural and eco-physiological traits of the Pugnose Shiner (*Miniellus anogenus*, formerly *Notropis anogenus*), a small leuciscid fish listed as Threatened under Canada’s Species at Risk Act (SARA), for which both elevated water temperature and turbidity have been identified as potential threats (COSEWIC, 2013; Gray *et al.*, 2014, 2016; McDonnell *et al.*, 2021).

Habitat degradation, land use change, urban development and extreme weather events can increase sedimentary and nutrient loading in freshwater ecosystems (Kemp *et al*., 2011; Wurtsbaugh *et al*., 2019). Larger quantities of inorganic particles in the water can increase sedimentary turbidity, while nutrients such as phosphorus and nitrogen can stimulate algal blooms and increase organic turbidity (Donohue and Garcia Molinos, 2009; Wurtsbaugh *et al.*, 2019). In turn, both sedimentary and organic turbidity can affect other abiotic factors such as water temperature or dissolved oxygen (DO) concentration. For example, deforestation of riparian habitats can increase both sedimentary runoff and solar radiation (Dudgeon *et al.*, 2006; Souza *et al.*, 2013). Increased sedimentary runoff can then result in larger quantities of suspended particles that absorb sunlight and warm up surrounding waters (Wotton, 1994; Kale, 2016). Warmer waters also stimulate harmful algal blooms that further spread hypoxic zones in freshwater ecosystems and increase organic turbidity (Utne-Palm, 2002; Ho and Michalak, 2020).

For fishes that typically inhabit clear-water habitats and rely on vision as a major sensory modality, increasing turbidity in freshwater ecosystems can modify their visual environment and affect multiple feeding and social behaviours (Donohue and Garcia Molinos, 2009). Suspended particles absorb and scatter light, reducing the contrast between objects and their background, which can lead to diminished visual range and poorer feeding performance in visual predators (Gardner, 1981; Utne-Palm, 2002; De Robertis *et al.*, 2003; Wellington *et al.*, 2010). Increased activity levels have also been observed in association with increased foraging efforts, perhaps to compensate for reduced visual range (Meager and Batty, 2007; Harvey and White, 2008). Impacts of high turbidity can scale to the community level since mating (Gray *et al.,* 2011), territorial (Berg and Northcote, 1985) and schooling behaviours (Kelley *et al.*, 2012; Gray *et al.*, 2014) can be altered as individuals allocate more time and energy to forage in turbid waters, and visual signalling between conspecifics is diminished. Sedimentary turbidity can also clog or damage gills, which may reduce oxygen uptake capacities of fish and other metabolically expensive activities such as swimming (Sutherland and Meyer, 2007; Gray *et al.*, 2014).

Elevated water temperature is increasingly recognized as a significant driver of variation in fish species and assemblages. Rising water temperatures have been associated with changes in fish distribution, behaviour and physiology (Strayer and Dudgeon, 2010; Reid *et al.*, 2019). As ectothermic vertebrates, the body temperature of fish is regulated by their environment, which can make them highly sensitive to elevated water temperatures and extreme thermal events (Seebacher *et al.*, 2015; Burraco *et al.*, 2020). The thermal performance window represents the range of temperatures across which fish can maintain vital functions such as activity, growth and reproduction (Huey and Stevenson, 1979; Schulte *et al.*, 2011). The lower and upper critical limits of this thermal performance window are referred to as critical thermal minimum (CT_min_) and critical thermal maximum (CT_max_), respectively. As environmental temperatures approach and eventually exceed the thermal limits of a species’ performance window, performance may decrease and death may occur if fish are unable to move to another habitat or adjust their thermal window via phenotypic plasticity and/or genetic change (Beitinger *et al.*, 2000; Pörtner, 2010; McDonnell and Chapman, 2015). In fishes, CT_max_ is often quantified as the temperature at which loss of equilibrium (LOE) occurs in response to an acute increase in water temperature (Lutterschmidt and Hutchison, 1997). There is also a growing number of studies that have detected a behavioural threshold that occurs prior to CT_max_ and may be ecologically relevant. These studies have observed that some fish exhibit an avoidance behaviour (denoted as quick and agitated swimming) at temperatures well below their CT_max_ (e.g. McDonnell and Chapman, 2015; Wells *et al.*, 2016; Turko *et al.*, 2020; Kochhann *et al.*, 2021). This agitation behaviour could be useful in escaping thermal stress, but it may also expose fish to predators and affect access to important resources (McDonnell and Chapman, 2015). Elevated water temperature is also associated with higher standard and maximal metabolic rates (Schulte, 2015; Neubauer and Andersen, 2019) and higher activity levels in fish (Biro *et al.*, 2010; Bartolini *et al.*, 2015), which may lead to compensatory responses such as an increase in foraging activity with higher predator encounter rates (Buentello *et al.*, 2000). With the persistent threat of global warming to freshwater fish biodiversity and the various stress responses induced by elevated water temperature, there is a growing need to understand how fishes respond not only to thermal stress but also how it interacts with other stressors.

Warming waters and high turbidity are likely to have interactive effects on fishes as they both have the potential to affect aerobic metabolism. For example, elevated water temperature can increase the rate of oxygen consumption; while high turbidity can reduce oxygen uptake efficiency by clogging gills with sedimentary particles or increasing stress levels through reduced visual range. A reduction in oxygen uptake efficiency could then make it more challenging to cope with heightened metabolic demands in warming waters and also impact fish behavioural and physiological responses to other environmental disturbances such as hypoxia (Fry, 1971; McBryan *et al.,* 2013; Claireaux and Chabot, 2016). Despite all these potential interactions between elevated water temperature and high turbidity, there are still few studies that examine the interactive effects of both stressors on freshwater fishes. We explore these potential interactions by quantifying the effects of water temperature and sedimentary turbidity on the swimming activity of Pugnose Shiner as well as their behavioural response to hypoxia and thermal tolerance.

The Pugnose Shiner has been listed as Threatened under Canada’s Species at Risk Act (SARA, 2019) since 2019 and under the Committee on the Status of Endangered Wildlife in Canada (COSEWIC) since 2013. The Canadian Pugnose Shiner populations currently represent the extreme northern range of this species (Chu *et al.*, 2005). Within Canada, a few small and isolated populations of Pugnose Shiner are found in Southern Ontario in highly vegetated streams and lakes with calm and clear water (< 2 nephelometric units (NTU); Gray *et al.*, 2014; McCusker *et al.*, 2014). In addition to nutrient loading and habitat degradation, turbidity has been proposed as one of the primary drivers of the decline in Canadian Pugnose Shiner (COSEWIC, 2013). Indeed, decreased swimming abilities, reduced schooling behaviour and lower hypoxia tolerance have been documented in Pugnose Shiner exposed to relatively low turbidity levels (< 10 NTU) compared to other closely related *Notropis* and *Miniellus* species (Gray *et al.*, 2014, 2016). Climate warming may also affect the persistence of Pugnose Shiner, as this species has been observed to have a lower CT_max_ and smaller thermal safety margin (difference between CT_max_ and acclimation temperature) than other non-imperiled *Notropis* and *Miniellus* species (McDonnell *et al.*, 2021).

To quantify the independent and interactive effects of water temperature and sedimentary turbidity on Pugnose Shiner, wild-caught fish were acclimated to clear and turbid water crossed with low and high water temperature. We measured their swimming activity in a group setting when exposed to a gradual increase in turbidity and during a long-term acclimation period. Following activity trials, we explored whether acclimation to high temperature and/or turbidity affected their response to hypoxia by quantifying agitation and aquatic surface respiration (ASR), where fish swim to the water surface and ventilate their gills in the oxygen rich layer (Chapman and Mckenzie, 2009). Measuring this behavioural response to hypoxia is ecologically relevant since ASR can increase predation risk and alter other important behaviours such as mating, competition and shoaling as fish spend more time at the water surface (Chapman and Mckenzie, 2009). After ASR trials, we finished our experiment by assessing the thermal tolerance of Pugnose Shiner with CT_max_ trials. An interaction between turbidity and temperature would be evident in this experiment if fish showed a different response to turbidity when acclimated to warm versus cool water. We predicted that fish acclimated to warm, turbid water would have a lower thermal tolerance than those acclimated to warm, clear water. This may reflect gill clogging from sedimentary particles and/or increased stress levels due to a reduced visual range combined with heightened metabolic demands potentially associated with an acute temperature increase during CT_max_ trials. We also predicted that fish acclimated to warm, turbid water would become agitated and use ASR at higher DO levels than fish acclimated to cool, clear water, again associated with reduced respiratory function in turbid water and higher metabolic demands of warm water.

## Materials and Methods

### Fish collection and experimental set-up

In October 2020, we live-captured Pugnose Shiner specimens from Thompson’s Bay in the upper St-Lawrence River area (between ‘44.408°N 75.908°W’ and ‘44.418°N 75.894°W’). Thompson’s Bay is one of the most important Pugnose Shiner habitats in Ontario with low turbidity levels (0.46 NTU at capture sites; Potts, 2020), high DO levels and abundant aquatic vegetation (McCusker *et al.,* 2014). We transported live specimens to McGill University and held them for 5 months in climate-controlled chambers at 16°C ± 0.01 (mean ± SEM) under a 12 h light:dark cycle. The water temperature of 16°C represented the average water temperature for September and October recorded in previous Pugnose Shiner surveys (Fisheries and Oceans Canada (DFO), unpublished data, 2002–2016). Fish were fed a daily diet of frozen blood worm *ad libitum*. In March 2021, we randomly distributed 95 Pugnose Shiner among nineteen 38-L aquaria (41 × 25 × 50 cm; height × width × length) with a density of 5 fish per aquarium. Each aquarium contained a filter-less water pump (Mini Underwater Filter, Fluval) that was filled with bio-balls to allow for the growth of nitrifying bacteria while minimizing the quantity of sedimentary particles removed from the water, an oxygen diffuser and plastic vegetation for environmental enrichment. Each aquarium was also separated from other aquaria by white plastic boards to prevent visual stimuli between adjacent aquaria. We monitored water temperature, DO, pH and conductivity daily throughout the entire experiment (Handy Polaris 2, Oxyguard; pH Testr10 and EC Testr11, Thermo Fisher Scientific) and performed necessary water changes (up to 10–20 L per day) to maintain water quality during the initial holding period. Aquaria were normoxic (DO > 95% saturation), pH levels were at 8.0 ± 0.0 and water conductivity was between 400 and 700 uS.

### Experimental treatments and exposure

Aquaria were randomly assigned to combinations of water temperature (16°C or 25°C) and turbidity (~0 NTU or 8.5 NTU) using a 2 × 2 crossed design. Since fish had been held at 16°C for 5 months in clear water, this experiment asked whether an increase in temperature and/or an increase in turbidity affected the Pugnose Shiner. Our elevated water temperature treatment was 2°C higher than the average water temperature recorded in July and August for sites inhabited by Pugnose Shiner in Canada (23°C; DFO, unpublished data, 2002–2016). This temperature was also selected to minimize the risk of mortality since McDonnell *et al.* (2021) acclimated Pugnose Shiner to water temperatures ranging from 16°C to 31°C and observed higher survivorship at temperatures between 16°C and 25°C with an increase in mortalities at 28°C and 31°C. Our turbid treatment of 8.5 NTU was selected to expose fish to turbid water while permitting visual observations of fish behaviour. This level was also close to the highest turbidity level used by Gray *et al.* (2014, 10 NTU) in their study on the effects of turbidity on social behaviours of Pugnose Shiner and other minnow species. Initially, each treatment combination had five replicate aquaria, except for the 16°C clear water treatment with only four replicates due to a limited number of specimens. Fish mortality per treatment was generally low, except for a few fish that did not survive fin clipping and tagging for pilot tests of a respirometry experiment. No further respirometry trials were attempted to avoid additional mortalities. All fish care, collection and experimental protocols were approved by McGill University Animal Care Committee (AUP 7951) and the following provincial and federal permits: Permit No. 20-PCAA-00030 (Fisheries and Oceans Canada), Permit No. ER-B-005-20 (Ministry of the Environment, Conservation and Parks, Ontario), License No. 1096064 (Ministry of Natural Resources and Forestry, Ontario), Permit No. 2020-07-06-2856-06-S-P (Ministère des Forêts, de la Faune et des Parcs, Quebec).

**Figure 1:**
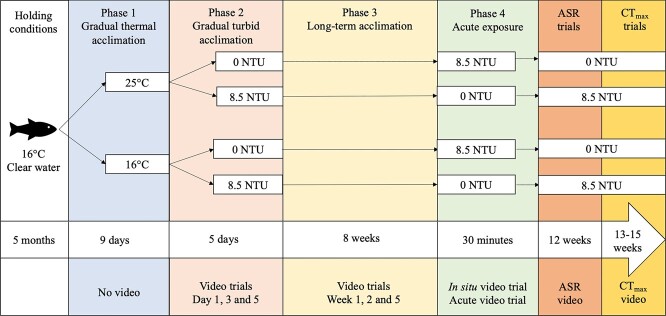
Experimental exposure timeline used to test for the independent and interactive effects of turbidity and temperature on Pugnose Shiner. Activity was measured over 8 weeks of acclimation (Phases 2 and 3) and during an acute exposure to the alternative turbidity condition (Phase 4; results not reported), ASR thresholds were measured after 12 weeks of acclimation, and thermal tolerance was measured with CT_max_ trials after 13–15 weeks of acclimation.

The experimental design consisted of three phases (Fig. 1). Phase 1 involved a gradual increase in water temperature (1°C/day) over 9 days for the 25°C thermal treatment using individual submersible aquarium heaters (Marina Submersible Heater, Hagen; McDonnell *et al.*, 2021; Potts *et al.*, 2021). During Phase 2, we gradually increased sedimentary turbidity over 5 days (1.4 NTU/day) for aquaria assigned to the 8.5 NTU turbidity treatment. Sedimentary turbidity was increased using a highly concentrated solution of natural bentonite clay powder similar to the one used in Gray *et al.* (2014, 2016, Healing Clay, Aztec Secret) mixed with water (30 g/L), while the ~ 0 NTU clear treatment had no clay added. We added a calculated amount of bentonite clay-water solution after any water change in turbid tanks to maintain the appropriate NTU level. We resuspended clay particles daily and measured turbidity after resuspension with a turbidity meter (2100Q Portable Turbidimeter, Hach). Phase 3 consisted of an 8-week long-term acclimation period to constant water temperatures (24.89 ± 0.01°C and 16.12 ± 0.01°C) and turbidities (0.79 ± 0.05 NTU and 8.57 ± 0.05 NTU) with daily monitoring. Following Phase 3, all fish also underwent an acute exposure to the alternative turbidity condition (30 min to either ~ 0 NTU or 8.5 NTU, results not reported here). After Phase 3 and the acute exposure, fish were acclimated for an additional 4 weeks before we measured aquatic surface respiration (ASR). Fish used in ASR trials were immediately euthanized and were not used in following critical thermal maximum (CT_max_) trials to control for any residual effects of hypoxia exposure (Ackerly *et al.,* 2018). For CT_max_ trials, all remaining fish were acclimated for an additional 1 to 3 weeks under the same temperature and turbidity conditions.

### Swimming activity trials

We recorded *in situ* videos using two cameras (Hero Session 4, GOPRO; Elite V50, AKASO) during Phase 2 (days 1, 3 and 5) and Phase 3 (weeks 1, 2, 5 and 8; Fig. 1). To measure fish activity, we traced a grid on the front of each aquarium to divide the viewing area into eight quadrats (12.5 × 7.5 cm, width × height). Following Gray *et al.* (2014), we placed a white plastic board in the aquarium to gently move the entire group of fish forward in the first half. We allowed fish to habituate for 30 min and then recorded movement over 5 min in the absence of any experimenter. Using the recorded videos, we then manually counted the number of grid quadrats visited over 5 min for each fish in the group before averaging activity data per aquarium to quantify swimming activity in a group setting. Only behavioural videos with a density of five fish were statistically analyzed due to mortalities in certain groups (tank sample size for Phase 2 and 3 reported in Table 1).

**Table 1:** Biometric data and sample size for the turbidity x temperature acclimation of Pugnose Shiner (see Fig. 1 for experimental design). For Phase 2 and 3, the number of tanks with a density of 5 fish is reported for each experimental treatment. For ASR and CT_max_ trials, the number of individual fish used is reported for each experimental treatment. Average mass (g) ± SEM as well as average standard and total length (cm) of fish are also reported per treatment. The mass and length values were only measured for fish that underwent ASR and CT_max_ trials and all values were averaged together.

Treatment	Phase 2 (# of tanks)	Phase 3 (# of tanks)	ASR (# of fish)	CT_max_ (# of fish)	Mass (g)	Standard length (cm)	Total length (cm)
16°C + 0 NTU	4	4	8	8	0.97 ± 0.05	4.33 ± 0.18	4.28 + 0.10
16°C + 8.5 NTU	5	4	8	9	1.00 ± 0.11	4.09 ± 0.17	4.40 ± 0.20
25 °C + 0 NTU	5	4	10	11	0.90 ± 0.04	4.34 ± 0.13	4.43 ± 0.12
25°C + 8.5 NTU	3	2	9	8	0.97 ± 0.05	4.34 ± 0.16	4.43 ± 0.14

### Aquatic surface respiration trials

To explore the effects of our acclimation conditions on fish response to hypoxia, we quantified the DO concentration at which fish started to become agitated (agitation threshold) and spent 90% of their time engaged in ASR behaviour (ASR90 threshold). ASR is a widespread behavioural response to hypoxia observed in many freshwater and marine fishes from both temperate and tropical ecosystems (reviewed in Chapman and McKenzie, 2009). In goldfish (*Carassius auratus*), ASR combined with bubble holding has been demonstrated to increase arterial blood oxygen content and therefore reduce the effects of aquatic hypoxia (Burggren, 1982). A higher ASR threshold can also be indicative of a lower tolerance to hypoxia because it is, in some species and/or populations, associated with a higher critical oxygen tension (P_crit_; oxygen level below which standard metabolic rate cannot be sustained; Chapman and McKenzie, 2009). After 12 weeks of acclimation, we conducted ASR trials on two Pugnose Shiner from each aquarium (*n* = 8–10 fish per treatment; Fig. 1; Table 1). Fish were not fed for 24 hours before the trial to induce a postabsorptive state. We transferred fish to individual baskets (13.5 × 15 × 12.5 cm) in a 40-L aquarium with thermal and turbidity conditions matching their acclimation conditions; and we allowed them to habituate for 2 hours. Bubble wrap covered half of the water surface to prevent oxygen diffusion, with half of the surface accessible to fish to perform ASR. We lowered DO% (percent air saturation) at a rate of 1% per min by bubbling nitrogen gas using a Witrox 1 unit, a DAQ-M instrument and the AutoResp software (Loligo Systems). DO% was lowered incrementally to levels of 100, 80, 70, 60, 50, 40, 30, 25, 20, 15, 10 and 5%. At every level, we maintained DO% constant over 5 min to allow us to record time-stamped videos, quantify agitation and ASR90 thresholds and count gill ventilations. ASR90 thresholds were measured as the DO% level at which fish spent 90% or more of their time performing ASR in the oxygen rich water surface. Agitation thresholds were measured as the DO% level at which fish started to swim with rapid bursts and occasional jumps for 30 s. Gill ventilation frequency was recorded for individual fish by averaging three ventilation counts, each of 15 s in length. For statistical analysis, gill ventilation frequency before and after the ASR90 threshold was also estimated for individual fish. Pre-ASR90 frequency was selected as the ventilation frequency recorded at the 40% DO level, a level above the ASR90 threshold of all experimental fish. Post-ASR90 frequency was selected as the ventilation frequency recorded at the 5% DO level, when all fish had crossed the ASR90 threshold. For presentation of the results, ventilation frequencies were converted to opercular beats per min (BPM). Once the three listed measurements were taken, DO% was further lowered until the next predetermined level. Trials ended at 5% DO or when fish lost equilibrium and fish were transferred to a fully aerated aquarium for recovery over 15 min. We then euthanized all fish used in ASR trials with eugenol and proceeded to weigh (g) and measure their total and standard length (cm).

### Critical thermal maximum trials

After 13–15 weeks of experimental acclimation, we conducted CT_max_ trials using all remaining fish (*n* = 8–11 fish per treatment; Fig. 1; Table 1). Fish were not fed for 24 hours before the trial to induce a postabsorptive state. We transferred fish to individual baskets (13.5 × 15 × 12.5 cm) in a 40-L aquarium with temperature and turbidity conditions matching their acclimation conditions; and we allowed them to habituate for 2 hours. The experimental aquarium had three baskets and each CT_max_ trial was conducted with one to three fish. We raised water temperature by 0.3°C/min using a thermal control system (Witrox 1 unit and DAQ-M instrument) and monitored DO% with the AutoResp software (Loligo Systems). We measured CT_max_ in Pugnose Shiner as the temperature at loss of equilibrium (LOE), which is when fish became unable to maintain an upright swimming position for 30 s. We also measured the agitation temperature (T_ag_) of Pugnose Shiner as the temperature at which fish started to swim with rapid bursts and occasional jumps for 30 s before LOE (McDonnell *et al.,* 2021; Potts *et al.,* 2021). We recorded time-stamped videos of all trials to quantify CT_max_ and T_ag_ temperatures. Using CT_max_ and T_ag_, two other thermal tolerance metrics were calculated. The agitation window was calculated as the difference between CT_max_ and T_ag_, as reported in Wells *et al.* (2016) and McDonnell *et al.* (2021). The thermal safety margin is generally calculated as the difference between CT_max_ and the maximum temperature recorded in the natural habitat of the fish (Morley *et al.*, 2019; Turko *et al.*, 2020). Here, we calculated a modified thermal safety margin as the difference between CT_max_ and the acclimation temperature (McArley *et al.*, 2017; McDonnell *et al.*, 2021). After CT_max_ trials, fish were transferred to a low water temperature aquarium for recovery over 15 min. We then euthanized, weighed (g) and measured the total and standard length (cm) of all fish used in CT_max_ trials.

### Analysis

To analyze swimming activity of fish in a group setting, we averaged individual activity counts per aquarium and only analyzed videos with a density of five fish. Phases 2 and 3 were analyzed separately. In Phase 2, only aquaria undergoing gradual turbidity exposure were included in the analysis since we were interested in the effects of a gradual increase in turbidity on fish activity. For Phase 3, we quantified the effects of chronic turbidity exposure on fish activity. For each phase, we used the glmer.nb() function to produce general linear mixed models (GLMM; package *lme4* v.1.1.30; Bates *et al.*, 2015). Based on the guidelines in Payne *et al.* (2018) for data overdispersion and a dispersion parameter of 6.31 and 3.45 for the Phase 2 and 3 models, respectively; we selected a negative binomial distribution rather than a Poisson distribution. We used the default error correlations in the glmer.nb() function for random and repeated measurement components (package *lme4* v.1.1.30; Bates *et al.*, 2015). For Phase 2, we used a GLMM to test for independent and interactive effects of water temperature (16°C or 25°C) and the acclimation day, which represented our gradually increasing turbidity condition, on average fish activity (fixed effects; sig. α < 0.05). For Phase 3, we used another GLMM to test for independent and interactive effects of water temperature (16°C or 25°C), turbidity (clear or turbid) and acclimation day on average fish activity (fixed effects; sig. α < 0.05). We added tank ID as a random effect in all models. All interactions terms were kept in the models even if non-significant because we were specifically interested in testing for an interaction between turbidity and temperature (R script in Supplementary Material). Wald chi-square values (Type III) are reported for all *P*-values of each model using the Anova() function (package *car* v.3.1–0; Fox and Weisberg, 2019). All statistical analyses were done using the R Studio software version 4.2.1. (R Core Team, 2022). GLMM outputs for fish activity during Phase 2 and 3 are summarized in our Supplementary Table S1.

For CT_max_ and ASR trials, we used the lmer() function to produce linear mixed models (LMMs; package *lme4* v.1.1.30; Bates *et al.*, 2015). With LMMs, we tested for independent and interactive effects of water temperature and turbidity (fixed effects; sig. α < 0.05) on fish ASR90 and agitation thresholds, CT_max_, T_ag_, agitation window and thermal safety margin. We added tank ID as a random effect in all models. Again, all interactions between temperature and turbidity were kept in the models even if non-significant because we were specifically interested in these interaction terms and their *P*-values, even if non-significant, can be informative (R script in Supplementary Material). For gill ventilation data, we used a GLMM to test for the effects of water temperature and turbidity (fixed effects; sig. α < 0.05) on gill ventilation frequency with tank ID as a random effect (R script in Supplementary Material). We also included individual fish as a repeated measure effect in this model since we compared pre-ASR90 and post-ASR90 gill ventilation frequency of the same fish to test if gill ventilation rates declined once fish crossed the ASR90 threshold. We selected a Poisson distribution for this GLMM since gill frequency was represented by discrete count data with a dispersion parameter of 0.41, resulting in relatively conservative *P*-values (Payne *et al.*, 2018). Again, we used the default error correlations in the lmer() and glmer() functions for random and repeated measure effects (package *lme4* v.1.1.30; Bates *et al.*, 2015). Fish mass and standard length were initially included as covariates, but removed from all models as they had no significant effect (α > 0.05). We performed multiple comparison post-hoc tests (Holm-Bonferroni method) among the different thermal and turbidity treatment combinations for all significant interactions (sig. α < 0.05; package *emmeans* v.1.8.1–1; Lenth, 2022). Wald chi-square values (Type III) are reported for all *P*-values of each model using the Anova() function (package *car* v.3.1–0; Fox and Weisberg, 2019). All LMM outputs for our ASR and CT_max_ trials and the GLMM output for our gill ventilation data are reported in our Supplementary Tables S2 and S3.

## Results

### Swimming activity trials

**Figure 2:**
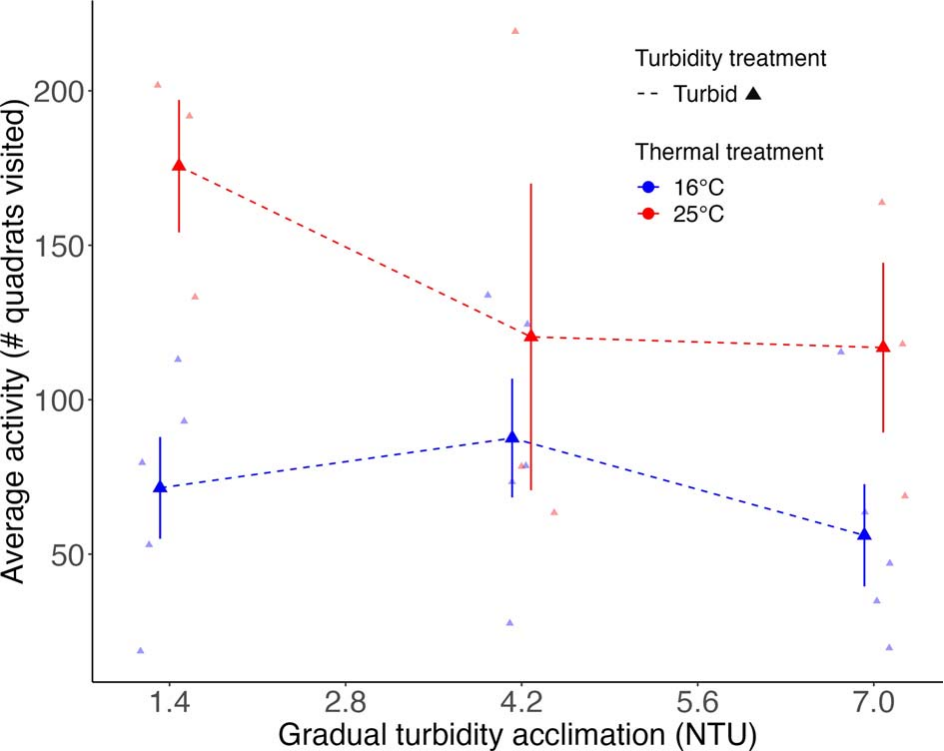
Swimming activity of Pugnose Shiner in a group setting under gradual turbidity increase over 5 days (Phase 2). Thermal treatment is indicated by colour (blue line at the bottom of the figure for 16°C and red line at the top of the figure for 25°C). Only aquaria undergoing gradual turbidity acclimation were analyzed (dashed lines and triangle points). Activity was measured as the total number of quadrats visited over 5 min per fish, and individual activity counts were averaged per aquarium. Larger points on the graph represent mean ± SEM while smaller points represent the raw data. Error bars and means in the figure are calculated using the raw data and are not corrected for the variance due to the aquarium random effect.

**Figure 3:**
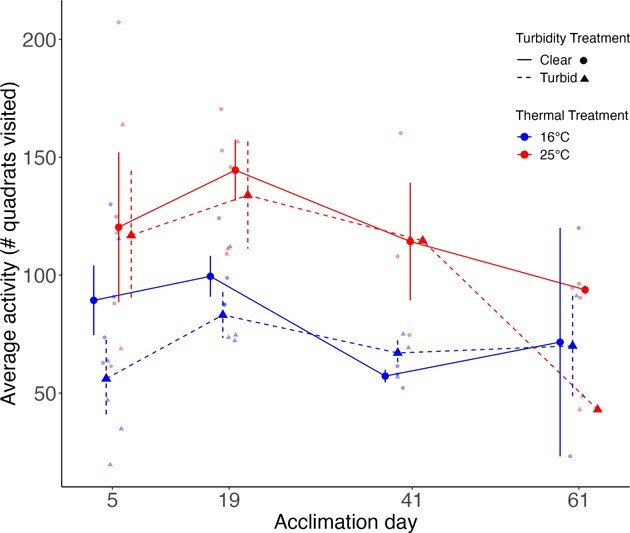
Swimming activity of Pugnose Shiner in a group setting during Phase 3 (8-week acclimation). Thermal treatment is indicated by colour (blue lines at the bottom of the figure for 16°C and red lines at the top of the figure for 25°C), and turbidity treatment is indicated by line and shape type (solid and circles for ~ 0 NTU (clear water) and dashed and triangles for 8.5 NTU (turbid water)). Larger points on the graph represent mean ± SEM while smaller points represent the raw data. Absence of error bars for the swimming activity recorded on days 41 and 61 for the turbid, warm treatment indicates that only one aquarium had a density of 5 fish at the end of Phase 3 due to fish mortality. Error bars and means in the figure are calculated using the raw data and are not corrected for the variance due to the aquarium random effect.

Overall, acclimation to warm water resulted in an increase in swimming activity of Pugnose Shiner (Figs 2 and 3). During Phase 2, fish in warm and cool water were exposed to increasing turbidity over a 5-day period. Pugnose Shiner were more active in warm water (25°C) than in cool water (16°C; χ^2^ = 4.22 *P* = 0.04), but there was no evidence of activity changing as days progressed and turbidity increased (χ^2^ = 1.09, *P* = 0.30; Fig. 2). During Phase 3, fish acclimated to warm water were more active than fish acclimated to cool water throughout the 8-week period of long-term acclimation (χ^2^ = 11.45, *P* = 0.0007; Fig. 3). However, there was no significant effect of turbidity (χ^2^ = 1.00, *P* = 0.32) or acclimation day (χ^2^ = 0.0005, *P* = 0.98), nor was there a significant interaction between temperature and turbidity (χ^2^ = 0.02, *P* = 0.89).

### Aquatic surface respiration trials

**Figure 4:**
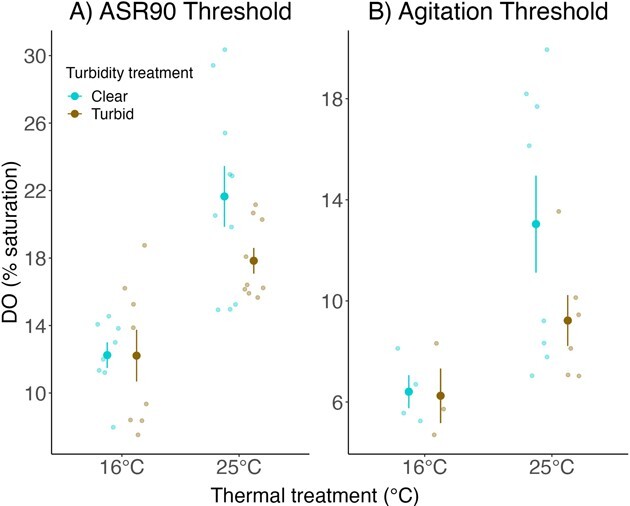
ASR90 thresholds (A) and agitation thresholds (B) of Pugnose Shiner after a 12-week acclimation period. Water temperature during the acclimation period is indicated on the x-axis and turbidity is indicated by colour (blue for clear water (~ 0 NTU) and brown for turbid water (8.5 NTU)). Larger points on the graph represent mean ± SEM while smaller points represent the raw data. Error bars and means in the figure are calculated using the raw data and are not corrected for the variance due to the aquarium random effect.

**Figure 5:**
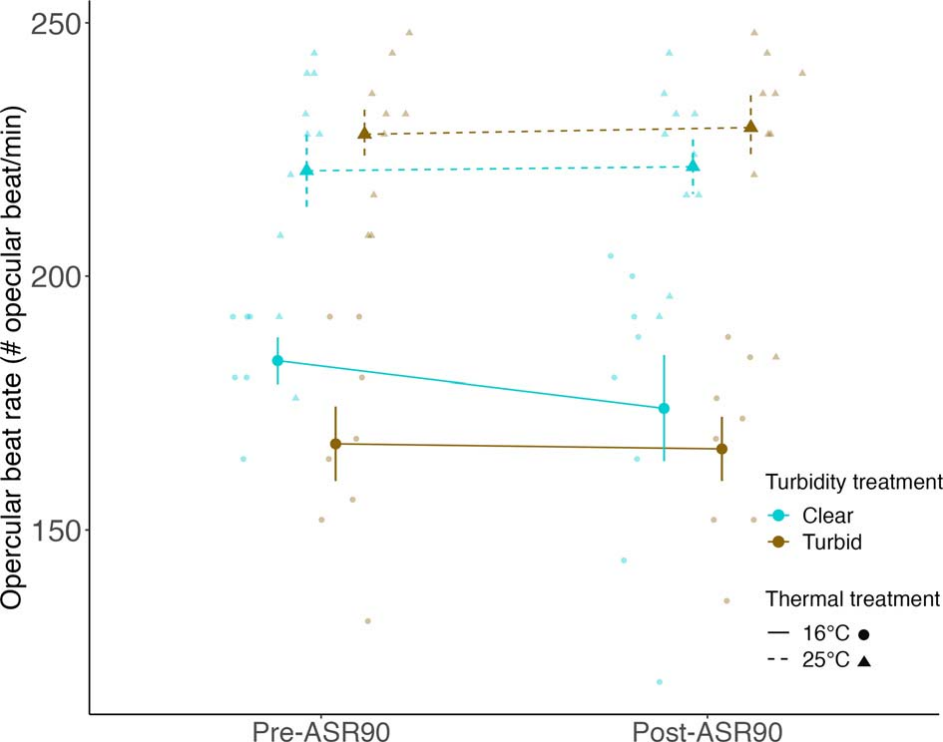
Opercular BPM (beat/min) of Pugnose Shiner pre-ASR90 and post-ASR90 thresholds. Pre-ASR90 BPM is the rate recorded at 40% DO, a level above the ASR90 threshold of all experimental fish and post-ASR90 BPM is the rate recorded at 5% DO, when all fish had crossed the ASR90 threshold. Water temperature is indicated by line and shape type (solid and circles for 16°C and dashed and triangles for 25°C) and turbidity is indicated by colour (blue for clear water (~ 0 NTU) and brown for turbid water (8.5 NTU)). Larger points on the graph represent mean ± SEM while smaller points represent the raw data. Error bars and means in the figure are calculated using the raw data and are not corrected for the variance due to the aquarium random effect.

Pugnose Shiner acclimated to warm water for 12 weeks exhibited higher ASR90 thresholds (mean ASR90 threshold = 19.85 ± 1.08% DO; mean ± SEM) than fish acclimated to cool water (mean ASR90 threshold = 12.23 ± 0.82% DO; χ^2^ = 19.87, *P* < 0.0001; Fig. 4A). Similarly, the DO% level at which fish became agitated in response to progressive hypoxia was higher in Pugnose Shiner acclimated to warm water (mean agitation threshold = 11.13 ± 1.25% DO) than in fish acclimated to cool water (mean agitation threshold = 6.33 ± 0.54% DO; χ^2^ = 8.04, *P* = 0.0045; Fig. 4B). We did not detect an effect of turbidity on ASR90 thresholds (χ^2^ = 0.0005, *P* = 0.98) or a significant interaction between temperature and turbidity (χ^2^ = 1.52, *P* = 0.22). Similarly, we did not detect an effect of turbidity on agitation thresholds (χ^2^ = 0.003, *P* = 0.96) or a significant interaction between temperature and turbidity (χ^2^ = 1.05, *P* = 0.31). Finally, Pugnose Shiner acclimated to warm water exhibited a higher opercular BPM than fish acclimated to cool water both pre-ASR90 and post-ASR90 thresholds (χ^2^ = 15.47, *P* < 0.0001; Fig. 5), while acclimation to turbidity had no effect (χ^2^ = 1.22, *P* = 0.27), nor was there a significant interaction between temperature and turbidity (χ^2^ = 1.60, *P* = 0.21).

### Critical thermal maximum trials

**Figure 6:**
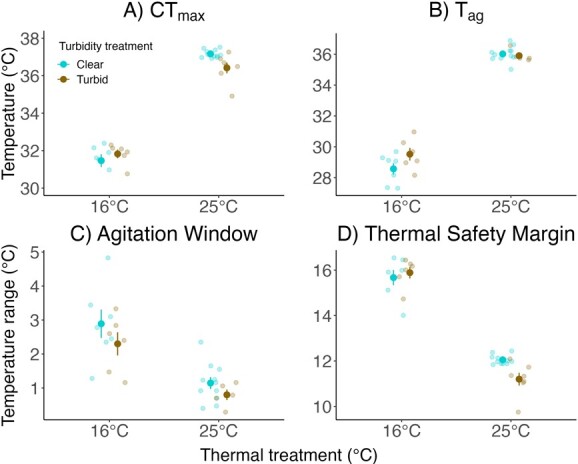
CT_max_ (A), T_ag_ (B), agitation window (C) and thermal safety margin (D) of Pugnose Shiner after a 13 to 15-week acclimation period. Water temperature used during the acclimation period is indicated on the x-axis, and turbidity is indicated by colour (blue for clear water (~ 0 NTU) and brown for turbid water (8.5 NTU)). Larger points on the graph represent mean ± SEM while smaller points represent the raw data. Error bars and means in the figure are calculated using the raw data and are not corrected for the variance due to the aquarium random effect.

During CT_max_ trials, Pugnose Shiner acclimated to warm water became agitated and lost equilibrium at higher temperatures than fish acclimated to cool water (χ^2^ = 357.72, *P* < 0.0001 for CT_max_ and χ^2^ = 277.01, *P* < 0.0001 for T_ag_). Additionally, there was a significant interaction between temperature and turbidity for CT_max_ (χ^2^ = 5.86, *P* = 0.016; Fig. 6A). CT_max_ was lower in fish acclimated to warm, turbid water (36.42 ± 0.28°C) than in fish acclimated to warm, clear water (37.16± 0.06°C; *P* < 0.0001; post-hoc). However, when fish were acclimated to cool water, there was no difference in CT_max_ between clear or turbid treatments (31.63 ± 0.21°C; *P* > 0.05; post-hoc). Agitation temperature (T_ag_) was much higher in fish acclimated to warm water (35.96 ± 0.11°C) than in fish acclimated to cool water (29.05 ± 0.29°C; χ^2^ = 277.01, *P* < 0.0001; Fig. 6B). There was no evidence that acclimation to turbidity affected T_ag_ (χ^2^ = 3.36, *P* = 0.07), and there was no significant interaction between turbidity and temperature (χ^2^ = 2.21, *P* = 0.14).

Pugnose Shiner in warm water had smaller agitation windows than fish in cool water (2.62 ± 0.27°C at 16°C and 1.04 ± 0.13°C at 25°C; Fig. 6C; χ^2^ = 22.08, *P* < 0.0001) with no significant effect of turbidity (χ^2^ = 1.92, *P* = 0.17) and no significant interaction between temperature and turbidity (χ^2^ = 0.17, *P* = 0.69). The thermal safety margin was much smaller in fish acclimated to warm water than in fish acclimated to cold water (χ^2^ = 142.26, *P* < 0.0001; Fig. 6D). There was no significant independent effect of turbidity (χ^2^ = 0.39, *P* = 0.54), but there was a significant interaction between turbidity and water temperature (χ^2^ = 5.42, *P* = 0.02). For fish acclimated to warm water, the thermal safety margin declined from 12.05 ± 0.06°C at ~ 0 NTU to 11.2 ± 0.28°C at 8.5 NTU (*P* < 0.0001; post-hoc), while fish acclimated to cold water showed an average thermal safety margin of 15.78 ± 0.21°C.

## Discussion

Swimming activity, aquatic surface respiration (ASR) and critical thermal maximum (CT_max_) trials revealed multiple effects of elevated water temperature and turbidity on Pugnose Shiner behaviour and physiology. Acclimation to warm water (25°C) increased fish activity and thermal tolerance (CT_max_ and T_ag_). However, fish acclimated to warm water also showed an earlier response to a gradual decrease in DO levels as well as a smaller agitation window and thermal safety margin than fish acclimated to cool water. Furthermore, fish acclimated to warm, turbid water exhibited a lower CT_max_ and had smaller thermal safety margin than fish acclimated to warm, clear water. We discuss the observed patterns in relation to the multiple stressor literature on the effects of turbidity, hypoxia and thermal stress, as well as the conservation implications of our results for the imperilled Pugnose Shiner.

### Swimming activity trials

Pugnose Shiner acclimated to warm water were more active than fish acclimated to cool water, while acclimation to turbidity had no effect on fish activity. Increased activity was an immediate response to high water temperature that did not vary significantly over time. This is consistent with previous studies where activity increased when fish were reared and/or acclimated to warmer temperatures (e.g. Biro *et al.*, 2010; Bartolini *et al.*, 2015; Colchen *et al.*, 2017; McDonnell *et al.*, 2019). Since higher temperatures have been associated with higher metabolic rates, fish in warmer water may show higher activity levels because of an increased metabolism (Bartolini *et al.*, 2015; Neubauer and Andersen, 2019). Fish in warm water were also more active throughout the long-term acclimation period and acclimation time did not significantly affect fish activity level. This might indicate that fish were unable to lower their standard metabolic rate through thermal compensation over the acclimation period. It is to be noted that our repeated measurements of activity over a long-term period may reflect fish acclimation to the experimental arena based on the overall decreasing trends shown in our Figs 2 and 3. However, this decrease in activity may have been missed due to the limited sample size in our study. Activity generally represents a large proportion of fish energy budgets, meaning that higher activity levels could reduce other energy intensive functions (Rennie *et al.*, 2005). Therefore, wild populations of Pugnose Shiner under continuous thermal stress may exhibit reduced growth and reproduction over time if activity levels remain heightened.

Turbidity had no independent or interactive effect on Pugnose Shiner activity. The absence of any turbidity effect could reflect the relatively low turbidity level used in our experiment (8.5 NTU), which was necessary to allow us to visually record fish behaviour. Gray *et al.* (2014) reported reduced school cohesion of Pugnose Shiner acclimated to relatively low turbidity levels (< 10 NTU), which suggests an impact of even low levels of turbidity on schooling behaviour. However, this response may not necessarily be captured in average swimming activity of fish in a group setting. It would be ecologically relevant to explore the effects of higher turbidity levels or acute turbidity exposure on the activity of Pugnose Shiner to mimic the effects of rainy season runoff or flooding events (Gray *et al.*, 2011).

### Aquatic surface respiration trials

Pugnose Shiner acclimated to warm water became agitated and used ASR at higher DO levels than fish in cool water, suggesting less tolerance to hypoxia. This effect of temperature on hypoxia tolerance, where hypoxia tolerance has been measured as critical oxygen tension (P_crit_; oxygen level below which standard metabolic rate cannot be sustained), ASR thresholds and/or LOE, has been documented in multiple fish species (e.g. Remen *et al.*, 2013; He *et al.*, 2015; Jung *et al.*, 2020; McArley *et al.*, 2020). The potential effects of elevated water temperature on hypoxia tolerance have also been explored in the context of the oxygen- and capacity-limited thermal tolerance (OCLTT) framework (Pörtner and Farrell, 2008; Pörtner, 2010). According to the OCLTT, fish maximal metabolic rate increases with temperature until the oxygen demands exceed the capacity of the cardiovacular system to provide oxygen to tissues (Pörtner, 2010). As a result, fish aerobic scope (difference between the maximal and standard metabolic rate) and performance may decrease (Pörtner, 2010). Consistent with the OCLTT is the idea that fish upper thermal limits should be highly sensitive to aquatic hypoxia since this stressor directly limits the amount of oxygen available for aerobic metabolism (Holeton, 1980; Pörtner, 2010; McBryan *et al.*, 2013). Similarly, fish hypoxia tolerance may be reduced under high water temperature conditions that increase oxygen demands (McBryan *et al.*, 2013). In Pugnose Shiner, previous studies have shown reduced thermal tolerance of juveniles and adults acclimated or acutely exposed to reduced oxygen levels (McDonnell *et al.*, 2021; Potts *et al.*, 2021). In our study, we explored the effects of chronic exposure to elevated water temperature on ASR behaviour. The increase in the ASR90 and agitation threshold in fish acclimated to high water temperature supports a thermal dependency of this response to hypoxia. Together with earlier studies on the Pugnose Shiner, there is increasing evidence for links between hypoxia and thermal tolerance in this imperilled fish. Future studies on the effects of elevated water temperature on Pugnose Shiner should integrate P_crit_ as a more direct evaluation of physiological responses to hypoxia under different thermal and turbid environments since it may yield informative and potentially different results compared to a behavioural response such as ASR.

Pugnose Shiner acclimated to warm water also exhibited a higher gill ventilation frequency (or opercular beats per min; BPM) than fish acclimated to cool water, potentially reflecting the increased rate of oxygen consumption associated with higher metabolic rates. Furthermore, fish in warm water maintained higher rates of gill ventilation throughout the ASR trials and engagement in ASR behaviour did not reduce gill ventilation rates, suggesting a relatively low efficiency of ASR in this species. In some fish species, the onset of ASR can reduce gill ventilation rates, likely due to an increase in oxygen uptake (Chapman and Liem, 1995). Pugnose Shiner exposed to thermal stress may then have engaged in ASR at higher oxygen concentrations and with higher gill ventilation frequencies to fufill greater metabolic demands and delay performance reduction (Holeton, 1980; McBryan *et al.*, 2013).

Turbidity showed no significant effect on Pugnose Shiner ASR behaviour. In addition, gill ventilation frequency was similar between fish acclimated to clear or turbid water. An earlier study on Pugnose Shiner reported a higher P_crit_ in fish acclimated to low levels of turbidity, which is consistent with the idea that turbidity may clog fish gills and reduce oxygen uptake efficiency (Gray *et al.,* 2016). Gills were not examined post-ASR trials, but Gray *et al.* (2016) reported a buildup of grey mucus on gill lamellae after acclimating Pugnose Shiner to 7 NTU for 3 to 6 months. Thus, Pugnose Shiner acclimated to 8.5 NTU for 3 months in our study could have also experienced a mucus buildup on their gills. Pugnose Shiner also had access to the water surface during ASR trials, whereas *P*_crit_ is measured in a closed system. Therefore, it is possible that access to the water surface compensated for any respiratory impairment due to high turbidity. Again, future studies measuring a physiological response to hypoxia such as *P*_crit_ or LOE could show an interactive or independent effect of a low turbidity level such as the one used in this experiment.

### Critical thermal maximum trials

Pugnose Shiner acclimated to warm water exhibited a higher CT_max_ and T_ag_ than fish acclimated to cool water, a pattern that has been previously observed in this species (McDonnell *et al.*, 2021; Potts *et al.*, 2021) and in several other fish species (reviewed in Beitinger *et al.*, 2000; Comte and Olden, 2017; Morley *et al.*, 2019). Despite evidence for thermal acclimation capacities, Pugnose Shiner acclimated to warm water had a smaller thermal safety margin than fish acclimated to cool water. This is consistent with previous studies on multiple freshwater fish species including Pugnose Shiner (Campos *et al.*, 2021; McDonnell *et al.*, 2021; Potts *et al.*, 2021). Therefore, wild populations of Pugnose Shiner in warm waters might have smaller thermal buffers against environmental warming compared to populations found in cooler waters. Fish in warm water also had smaller agitation windows, which can allow them to delay agitation and maintain normal behaviours for longer in warming waters. However, fish would potentially have less time to escape extreme thermal events in their environment before losing equilibrium. In addition, there is evidence that Pugnose Shiner may be more sensitive to thermal stress than other closely related species. McDonnell *et al.* (2021) quantified the CT_max_ of Pugnose Shiner across several acclimation temperatures. Using literature-derived data on other congeners, they found that Pugnose Shiner exhibited a lower CT_max_ and smaller thermal safety margins than non-imperilled congeners under comparable acclimation conditions.

Acclimation to turbidity decreased CT_max_ and the thermal safety margin of Pugnose Shiner acclimated to warm water. This reduced thermal tolerance could be due to a higher stress level in fish exposed to sedimentary turbidity that limited visual range and altered social behaviours and/or clogging of the gills that diminished oxygen uptake efficiency while oxygen demands increased with temperature. The turbidity level used in our study was well above the mean turbidity level (0.46 NTU) measured in Thompson’s Bay (site of specimen collection; Potts, 2020), but lower than the maximum observed at the same site (12.5 NTU; Potts, 2020). Although there was only a small decrease in CT_max_ of fish acclimated to warm, turbid waters, our data supports several reports over the years that Pugnose Shiner is sensitive to turbidity (Edwards *et al.*, 2012), and that effects can be observed at low levels of sedimentary loading, an observation also supported by the studies of Gray *et al.* (2014, 2016).

### Limitations

The absence of turbidity effect in behavioural metrics such as swimming activity, ASR thresholds and agitation temperatures may be due to variability among individuals in association with different personalities or other phenotypic traits. These behavioural metrics may then be underpowered compared to a physiological threshold such as CT_max_, for which we observed an interactive effect between temperature and turbidity. Furthermore, a limited sample size had to be used throughout the experiment to minimize the impact of our study on the natural population of Thompson’s Bay, which may have affected the power of our statistical analysis of the behavioural parameters measured. It is also possible that exposing fish to greater turbidity levels that are still ecologically relevant or using another turbidity medium other than bentonite clay could show different results. Nonetheless, it remains concerning that we observed an interactive effect of turbidity on fish thermal tolerance at a relatively low level, one that is already under the maximum turbidity value recorded in some natural Pugnose Shiner habitats. Therefore, the potential effects of sedimentary turbidity on Pugnose Shiner should not be disregarded without further studies on other behavioural and physiological metrics that play important roles in the survival and health of this species.

## Conclusion

The mechanisms underlying the effects of multiple stressors on fishes remain difficult to understand as freshwater ecosystems continue to undergo severe anthropogenic disturbances and climate variability. Isolated populations of imperilled fish with limited range expansion capacities will have to either adapt *in situ* to these disturbances or continue to decline. To mitigate population decline, species repatriation has been suggested as a potential conservation action for multiple imperilled fish species, including Pugnose Shiner (Edwards *et al.*, 2012; Lamothe *et al.*, 2019). This strategy has been applied with success in Chaumont Bay (Lake Ontario; New York), where thousands of pond-reared Pugnose Shiner have been released to restore the isolated population in the area (Foster *et al.*, 2021). However, knowledge gaps still surround the biology and ecology of Pugnose Shiner that might diminish the efficiency of such conservation strategy over the long-term. COSEWIC (2013) has listed turbidity and sediment loading as one of the main threats for this species in addition to habitat loss and degradation. Our study shows that relatively low levels of sedimentary turbidity decreased the thermal tolerance of this species under warm water conditions. Future conservation strategies should then combine actions to reduce sediment loading in critical habitats for Pugnose Shiner with pond-rearing techniques to improve the long-term success of repatriation efforts and increase the protection of extant populations. Furthermore, other threats listed by COSEWIC (2013) such as loss of aquatic vegetation, nutrient loading and algal blooms, range shifts of predators and invasive species may interact with sedimentary turbidity and result in severe impacts on Pugnose Shiner behaviour and physiology. Therefore, our study highlights the importance of exploring potential interactions between stressors when evaluating threats to imperilled species and potential recovery strategies.

## Supplementary Material

Web_Material_coae053

## Data Availability

Supplementary material, datasets and R script codes underlying this article are publicly available from Figshare at https://doi.org/10.6084/m9.figshare.25180445
