# Supplementary material for: Interactive effects of sedimentary turbidity and elevated water temperature on the Pugnose Shiner (Miniellus anogenus), a threatened freshwater fish
Source: Conserv Physiol. 2024 Aug 13;12(1):coae053. doi: 10.1093/conphys/coae053 (PMC11320368; doi:10.1093/conphys/coae053)
Supplement: Web_Material_coae053 [file web_material_coae053.zip › Supplementary_Material.pdf]

Table 1: Final general linear mixed models (GLMM) for phase 2 and 3, testing the effects of temperature (16 °C and 25 °C), turbidity (clear and turbid), and acclimation day on Pugnose Shiner swimming activity (package *lme4* v.1.1.30).

Activity: Phase 2

*glmer.nb(rounded.activity ~ temp.dis * day + (day | tank.number)*

Fixed effect	Estimate	SE	z value
(Intercept)	4.43	0.25	17.86
25 °C	0.79	0.38	2.06
Acclimation day	-0.08	0.07	-1.04
25 °C : Acclimation day	-0.04	0.11	-0.34
**Random effect**	**Name**	**Variance**	**SD**
Tank	(Intercept)	0.026	0.16
Acclimation day		0.002	0.04

Activity: Phase 3

*glmer.nb(rounded.activity ~ temp.dis * turb.dis + day + (day | tank.number)*

Fixed effect	Estimate	SE	z value
(Intercept)	4.40	0.15	29.31
25 °C	0.42	0.13	3.38
Turbid	-0.14	0.14	-1.00
Acclimation day	-0.0001	0.005	-0.02
25 °C : Turbid	0.03	0.22	0.13
**Random effect**	**Name**	**Variance**	**SD**
Tank	(Intercept)	0.14	0.38

Table 2: Final mixed models (LMM and GLMM) for hypoxia tolerance trials, testing the effects of temperature and turbidity on Pugnose Shiner ASR90 thresholds, agitation thresholds, and gill ventilation frequency (package *lme4* v.1.1.30).

ASR90 thresholds

*lmer(asr.90 ~ temp.dis * turb.dis + (1 | tank.number)*

Fixed effect	Estimate	SE	t value
(Intercept)	12.25	1.57	7.79
25 °C	9.40	2.11	4.46
Turbid	-0.05	2.20	-0.02
25 °C : Turbid	-3.68	2.99	-1.23
**Random effect**	**Name**	**Variance**	**SD**
Tank	(Intercept)	3.51	1.87

Agitation thresholds

*lmer(agitation ~ temp.dis * turb.dis + (1 | tank.number)*

Fixed effect	Estimate	SE	t value
(Intercept)	6.41	1.91	3.36
25 °C	6.63	2.34	2.84
Turbid	-0.16	2.92	-0.06
25 °C : Turbid	-3.66	3.57	-1.02
**Random effect**	**Name**	**Variance**	**SD**
Tank	(Intercept)	0	0

Ventilation Frequency

*glmer(vf ~ pre.post + temp.dis * turb.dis + (pre.post | tank.number),family = 'poisson')*

Fixed effect	Estimate	SE	z value
(Intercept)	3.80	0.05	77.62
Post-ASR90 threshold	-0.01	0.03	-0.19
25 °C	0.22	0.56	3.93
Turbid	-0.07	0.06	-1.11
25 °C : Turbid	0.03	0.08	1.26
**Random effect**	**Name**	**Variance**	**SD**

Tank	(Intercept)	0.001	0.03
Post-ASR90 threshold		0.00002	0.01

Table 3: Final linear mixed models (LMM) for thermal tolerance trials, testing the effects of temperature and turbidity on Pugnose Shiner CT_{max}, T_{ag}, agitation windows and thermal safety margins (package *lme4* v.1.1.30).

CT_{max}

*lmer(ctmax ~ temp.dis * turb.dis + (1 | tank.number)*

Fixed effect	Estimate	SE	t value
(Intercept)	31.47	0.24	133.59
25 °C	5.70	0.30	18.91
Turbid	0.36	0.35	1.04
25 °C : Turbid	-1.11	0.46	-2.42
**Random effect**	**Name**	**Variance**	**SD**
Tank	(Intercept)	0.005	0.07

T_{ag}

*lmer(tag ~ temp.dis * turb.dis + (1 | tank.number)*

Fixed effect	Estimate	SE	t value
(Intercept)	28.62	0.38	82.45
25 °C	7.36	0.44	16.64
Turbid	0.91	0.49	1.83
25 °C : Turbid	-1.02	0.68	-1.49
**Random effect**	**Name**	**Variance**	**SD**
Tank	(Intercept)	0.18	0.42

Agitation window

*lmer(window ~ temp.dis * turb.dis + (1 | tank.number)*

Fixed effect	Estimate	SE	t value
(Intercept)	2.89	0.29	9.97
25 °C	-1.74	0.37	-4.70
Turbid	-0.59	0.43	-1.39
25 °C : Turbid	0.24	0.59	0.41
**Random effect**	**Name**	**Variance**	**SD**
Tank	(Intercept)	0.008	0.09

Thermal safety margin<br>*lmer(margin ~ temp.dis * turb.dis + (1 | tank.number)*			
Fixed effect	Estimate	SE	t value
(Intercept)	15.67	0.24	66.0
25 °C	-3.62	0.30	-11.93
Turbid	0.22	0.35	0.62
25 °C : Turbid	-1.07	0.46	-2.33
Random effect	Name	Variance	SD
Tank	(Intercept)	0.009	0.10

```
# PUGNOSE SHINER ACTIVITY ANALYSIS

# DOWNLOAD DATASET
avg.activity.df <- read_csv2("DATA/avg.activity.csv",
                             na = "empty",
                             trim_ws = TRUE)

#### Phase 2 analysis (gradual turbidity increase) ####

# CREATE PHASE 2 DATASET
activity.phase2.df <- subset(avg.activity.df,
                             day != “A” & day != “19” & day != “40” & day !=
                             "61" & turb.dis != "C" & tank.number !="5" &
                             tank.number !="17")

# Removed tanks 5 and 17 since they only have activity data for day 1
# Removed days after day 5 (last day of gradual turbid acclimation)
# Removed tanks with clear water (only turbid tanks were analyzed)

# ADJUST DATA STRUCTURE
activity.phase2.df$turb.dis <- as.factor(activity.phase2.df$turb.dis)
activity.phase2.df$temp.dis <- as.factor(activity.phase2.df$temp.dis)
activity.phase2.df$day <- as.numeric(activity.phase2.df$day)
activity.phase2.df$tank.number <- as.factor(activity.phase2.df$tank.number)

# CREATE MEAN, SD AND STANDARD ERROR DATA
activity.phase2.df <- activity.phase2.df %>%
                      group_by(treatment, day) %>%
                      mutate(mean.activity = mean(activity, na.rm = TRUE)) %>%
                      mutate(sd.activity = sd(activity, na.rm = TRUE)) %>%
                      mutate(se.activity = std.error(activity, na.rm = TRUE))
                     %>%
                      ungroup()

# CREATE GRAPH
phase2.graph <-ggplot(data = activity.phase2.df, aes(x = gradual.turb, y =
mean.activity, colour = temp.dis, linetype = turb.dis, shape = turb.dis)) +
                geom_point(aes(y= mean.activity),
                           size = 3,
                           position=position_dodge(width = 0.3))+
                geom_point(aes(y= activity),
                           alpha = 0.4,
                           position=position_jitterdodge(dodge.width = 0.3)) +
                labs(x = "Gradual turbidity acclimation (NTU)",
                     y = "Average activity (# quadrats visited)",
                     title = NULL, colour ="Thermal treatment",
                     linetype = "Turbidity treatment") +
                guides(shape = “none”) +
                theme_classic() +
                geom_errorbar(aes(ymin = mean.activity-se.activity,
                                  ymax = mean.activity+se.activity),
                              width = 0,
                              position = position_dodge(width = 0.3),
```

```
                         linetype = 1) +
              theme(plot.title = element_text(hjust = 0.5)) +
              theme(legend.position = c(0.75, 0.8)) +
              theme(legend.text = element_text(size = 15)) +
              theme(legend.title = element_text(size = 15)) +
              scale_shape_manual(values = 17) +
              scale_colour_manual(labels = c("16°C", "25°C"),
                                  values = c("blue", "red")) +
              theme(text = element_text(size = 20)) +
              geom_line(position = position_dodge(width = 0.3)) +
              scale_linetype_manual(labels = "Turbid", values = "dashed") +
              theme(axis.text = element_text(size = 20)) +
              scale_y_continuous(breaks = seq(from = 0, to = 300, by = 50)) +
              scale_x_continuous(breaks = seq(from = 0, to = 7, by = 1.4))

# CHECK ASSUMPTIONS FOR MODEL
hist(activity.phase2.df$activity)
qqnorm(activity.phase2.df$activity)
qqline(activity.phase2.df$activity)
# Activity seems normally distributed

table(activity.phase2.df[, c("treatment","day")])
# Uneven sample size for the two treatments

# CHECK IF NEED RANDOM EFFECT IN MODEL
activity.phase2.lm <- lm(activity ~ temp.dis + day,
                         data = activity.phase2.df)

avg.lm.test.resid <- rstandard(activity.phase2.lm)

plot(avg.lm.test.resid ~ as.factor(activity.phase2.df$tank.number),
     xlab = "tank number",
     ylab = "Standardized residuals")
abline(0, 0, lty = 2)
# Yes, this suggests a random effect of tank

# CREATE MODEL
activity.phase2.glmm <- glmer(rounded.activity ~ temp.dis * day + (day |
                          tank.number),
                          data = activity.phase2.df,
                          family = "poisson")

# Check for overdispersion when using Poisson distribution
dispersion_glmer(activity.phase2.glmm)^2
# Dispersion parameter = 6.31 so overdispersed data and tried with negative
binomial distribution

activity.phase2.glmm <- glmer.nb(rounded.activity ~ temp.dis * day + (day |
                        tank.number),
                        data = activity.phase2.df)

summary(activity.phase2.glmm)
Anova(activity.phase2.glmm, type = 3)
```

```
# Used Type III since design is unbalanced due to fish death

# CHECK MODEL VALIDITY
# Dispersion
dispersion_glmer(activity.phase2.glmm)^2
# Dispersion parameter = 1.04 so no overdispersion

# Homogeneity of variance
plot(resid(activity.phase2.glmm) ~ fitted(activity.phase2.glmm), xlab =
"Predicted values", ylab = "Normalized residuals")
abline(h = 0, lty = 2)

# Normality
qqnorm(resid(activity.phase2.glmm))
hist(resid(activity.phase2.glmm))
```

#### Phase 3 analysis (longterm acclimation) ####

```
# CREATE PHASE 3 DATASET
activity.phase3.df <- subset(avg.activity.df,
                             day != "A" & day != "1" & day != "3")
# Remove any acute exposure and phase 2

# ADJUST DATA STRUCTURE
activity.phase3.df$turb.dis <- as.factor(activity.phase3.df$turb.dis)
activity.phase3.df$temp.dis <- as.factor(activity.phase3.df$temp.dis)
activity.phase3.df$day <- as.numeric(activity.phase3.df$day)
activity.phase3.df$tank.number <- as.factor(activity.phase3.df$tank.number)

# CREATE MEAN AND STANDARD ERROR DATA
activity.phase3.df <- activity.phase3.df %>%
                      group_by(treatment, day) %>%
                      mutate(mean.activity = mean(activity, na.rm = TRUE)) %>%
                      mutate(sd.activity = sd(activity, na.rm = TRUE)) %>%
                      mutate(se.activity = std.error(activity, na.rm = TRUE))
                     %>%
                      ungroup()

# CREATE GRAPH
phase3.graph <- ggplot(data = activity.phase3.df,
                       aes(x = day,
                           y = mean.activity,
                           colour = temp.dis,
                           linetype = turb.dis,
                           shape = turb.dis)) +
                geom_point(size = 3,
                           position = position_dodge(width = 6)) +
                geom_line(position = position_dodge(width = 6)) +
                geom_errorbar(aes(ymin = mean.activity - se.activity,
                                  ymax = mean.activity + se.activity),
                              width = 0,
                              position = position_dodge(width = 6)) +
                labs(x = "Acclimation day",
```

```
                     y = "Average activity (# quadrants visited)",
                     colour = "Thermal Treatment",
                     linetype = "Turbidity Treatment",
                     title = NULL ) +
                guides(shape = "none") +
                theme_classic() +
                theme(plot.title = element_text(hjust = 0.5)) +
                theme(text = element_text(size = 20)) +
                theme(legend.position = c(0.9, 0.8)) +
                theme(legend.text = element_text(size=13)) +
                theme(legend.title = element_text(size=13)) +
                scale_colour_manual(labels = c("16°C", "25°C"),
                                    values = c("blue","red")) +
                scale_x_continuous(breaks = c(5,19,41,61)) +
                scale_linetype_manual(labels = c("Clear", "Turbid"),
                                      values = c("solid","dashed")) +
                theme(axis.text = element_text(size = 20)) +
                geom_point(aes(y=activity,
                               shape = turb.dis),
                           alpha = 0.4,
                           position = position_jitterdodge(dodge.width = 1))

# CHECK ASSUMPTIONS FOR MODEL
hist(activity.phase3.df$activity)
qqnorm(activity.phase3.df$activity)
qqline(activity.phase3.df$activity)
# Activity seems normally distributed

table(activity.phase3.df[, c("treatment","day")])
# Uneven sample size for the treatments

# CHECK IF NEED RANDOM EFFECT IN MODEL
activity.phase3.lm <- lm(activity ~ temp.dis + day,
                         data = activity.phase3.df)

avg.lm.test.resid <- rstandard(activity.phase3.lm)

plot(avg.lm.test.resid ~ as.factor(activity.phase3.df$tank.number),
     xlab = "tank number",
     ylab = "Standardized residuals")
abline(0, 0, lty = 2)
# Yes, this suggests a random effect of tank #

# CREATE MODEL

activity.phase3.glmm <- glmer(rounded.activity ~ temp.dis * turb.dis + day +
                              (day | tank.number),
                              data = activity.phase3.df,
                              family = "poisson")

# Check for overdispersion when using Poisson distribution
dispersion_glmer(activity.phase3.glmm)^2
```

```
# Dispersion parameter = 3.45 so overdispersed data and tried with negative
binomial distribution


activity.phase3.glmm <- glmer.nb(rounded.activity ~ temp.dis * turb.dis + day +
                                (day | tank.number),
                                data = activity.phase3.df,
                                control =
                                glmerControl(optimizer="Nelder_Mead"))

summary(activity.phase3.glmm)
Anova(activity.phase3.glmm, type = 3)

# CHECK MODEL VALIDITY
# Overdispersion
dispersion_glmer(activity.phase3.glmm)^2
# Dispersion parameter = 1.03 so no overdispersion

# Homogeneity of variance #
plot(resid(activity.phase3.glmm) ~ fitted(activity.phase3.glmm),
     xlab = "Predicted values",
     ylab = "Normalized residuals")
abline(h = 0, lty = 2)

# Normality #
qqnorm(resid(activity.phase3.glmm))
hist(resid(activity.phase3.glmm))
```

```
# PUGNOSE SHINER ASR AND VENTILATION FREQUENCY ANALYSIS

# DOWNLOAD DATASET
asr.df <- read_csv2("DATA/asr.csv",
                    na = "empty",
                    trim_ws = TRUE)

#### ASR AND AGITATION ####

# ADJUST DATA STRUCTURE
asr.df$temp.dis <- as.factor(asr.df$temp.dis)
asr.df$turb.dis <- as.factor(asr.df$turb.dis)
asr.df$tank.number <- as.factor(asr.df$tank.number)

# CREATE MEAN AND STANDARD ERROR DATA
asr.df <- asr.df %>%
          group_by(treatment) %>%
          mutate(mean.asr.90 = mean(asr.90, na.rm = TRUE)) %>%
          mutate(se.asr.90 = std.error(asr.90, na.rm = TRUE)) %>%
          mutate(sd.asr.90 = sd(asr.90, na.rm = TRUE)) %>%
          mutate(mean.agitation = mean(agitation, na.rm = TRUE)) %>%
          mutate(se.agitation = std.error(agitation, na.rm = TRUE)) %>%
          mutate(sd.agitation = sd(agitation, na.rm = TRUE)) %>%
          ungroup()

# CREATE GRAPH
asr90.graph <- ggplot(data = asr.df,
                      aes(x = temp.dis,
                          y = mean.asr.90,
                          colour = turb.dis)) +
               geom_point(size = 3,
                          position = position_dodge(width = 0.5)) +
               geom_point(aes(y = asr.90),
                          alpha = 0.4,
                          position = position_jitterdodge(dodge.width = 0.5)) +
               labs(x = NULL,
                    y = "DO (% saturation)",
                    title = "A) ASR90 Threshold",
                    colour = "Turbidity treatment") +
               theme_classic() +
               geom_errorbar(aes(ymin = mean.asr.90 - se.asr.90,
                             ymax = mean.asr.90 + se.asr.90),
                             width = 0 ,
                             position = position_dodge(width = 0.5)) +
               theme(plot.title = element_text(hjust = 0.5)) +
               scale_colour_manual(labels = c("Clear", "Turbid"),
                          values = c("darkturquoise", "darkgoldenrod4")) +
               theme(text = element_text(size = 20)) +
               scale_x_discrete(labels=c("16" = "16°C", "25" = "25°C")) +
               scale_y_continuous(breaks = seq(from = 6, to = 30, by = 4)) +
               theme(axis.text = element_text(size = 20)) +
               theme(legend.position = c(0.3, 0.85)) +
               theme(legend.text = element_text(size = 15)) +
```

```
                theme(legend.title = element_text(size = 15))

agitation.graph <- ggplot(data = asr.df,
                          aes(x = temp.dis,
                              y = mean.agitation,
                              colour = turb.dis)) +
                   geom_point(size = 3,
                              position = position_dodge(width = 0.5)) +
                   geom_point(aes(y = agitation),
                              alpha = 0.4,
                              position = position_jitterdodge(dodge.width =
                              0.5)) +
                   geom_point(size = 3,
                              position = position_dodge(width = 0.5)) +
                   labs(x = NULL,
                        y = NULL,
                        title = "B) Agitation Threshold",
                        colour = NULL) +
                   theme_classic() +
                   geom_errorbar(aes(ymin = mean.agitation - se.agitation,
                                     ymax = mean.agitation + se.agitation),
                                 width = 0,
                                 position = position_dodge(width = 0.5)) +
                   theme(plot.title = element_text(hjust = 0.5)) +
                         scale_colour_manual(values = c("darkturquoise",
                         "darkgoldenrod4"),
                         guide = "none") +
                   theme(text = element_text(size = 20)) +
                   theme(panel.grid.minor = element_blank()
                         ,panel.grid.major = element_blank()) +
                   scale_x_discrete(labels = c("16" = "16°C", "25" = "25°C")) +
                   scale_y_continuous(breaks = seq(from = 6, to = 30, by = 4))+
                   theme(axis.text = element_text(size = 20))

combined.asr.graph <- ggarrange(asr90.graph,
                                agitation.graph,
                                align = "h")

combined.asr.graph <- annotate_figure(combined.asr.graph,
                                      bottom = text_grob("Thermal treatment (°C)",
                                      color = "black",
                                      size = 20))

# CHECK ASSUMPTIONS FOR MODEL
# Normality
hist(asr.df$asr.90)
qqnorm(asr.df$asr.90)
qqline(asr.df$asr.90)
# ASR 90 is normally distributed

hist(asr.df$agitation)
qqnorm(asr.df$agitation)
qqline(asr.df$agitation)
```

```
# Homogeneity of variance assumption
leveneTest(asr.90~ treatment, data = asr.df)
leveneTest(agitation~ treatment, data = asr.df)
# Not homogeneous variance

table(asr.df[,"treatment"])
# Not equal data due to fish mortality

# CHECK IF NEED RANDOM EFFECT IN MODEL
asr.lm <- lm(asr.90 ~ temp.dis + turb.dis,
             data = asr.df)

avg.lm.test.resid <- rstandard(asr.lm)

plot(avg.lm.test.resid ~ as.factor(asr.df$tank.number),
     xlab = "tank number",
     ylab = "Standardized residuals")
abline(0, 0, lty = 2)
# Yes, this suggests a random effect of tank #

# CREATE MODEL
asr90.lmer <- lmer(asr.90 ~ temp.dis*turb.dis + ( 1 | tank.number),
                   data = asr.df)

summary(asr90.lmer)
Anova(asr90.lmer, type = 3)

agitation.lmer <- lmer(agitation ~ temp.dis*turb.dis + ( 1 | tank.number),
                       data = asr.df)

summary(agitation.lmer)
Anova(agitation.lmer, type = 3)

# CHECK MODEL VALIDATION
# Homogeneity of variance
plot(resid(asr90.lmer) ~ fitted(asr90.lmer), xlab = "Predicted values", ylab =
"Normalized residuals")
abline(h = 0, lty = 2)

plot(resid(agitation.lmer) ~ fitted(agitation.lmer), xlab = "Predicted values",
ylab = "Normalized residuals")
abline(h = 0, lty = 2)

# Normality
qqnorm(resid(asr90.lmer))
hist(resid(asr90.lmer))
densityPlot(resid(asr90.lmer))

qqnorm(resid(agitation.lmer))
hist(resid(agitation.lmer))
densityPlot(resid(agitation.lmer))
```

```
#### VENTILATION FREQUENCY/BPM ANALYSIS ####

# DOWNLOAD DATASET
vf.df <- read_csv2("DATA/vf.csv",
                   na = "empty",
                   trim_ws = TRUE)

# ADJUST DATA STRUCTURE
vf.df$temp.dis <- as.factor(vf.df$temp.dis)
vf.df$tank.number <- as.factor(vf.df$tank.number)
vf.df$turb.dis <- as.factor(vf.df$turb.dis)
vf.df$pre.post <- factor(vf.df$pre.post, levels = c("pre", "post"))

# CREATE BPM DATA (VF values x 4 for graph)
vf.df$bpm <- vf.df$vf * 4

# CREATE MEAN, SD AND STANDARD ERROR DATA FOR BPM
vf.df <- vf.df %>%
         group_by(treatment, pre.post) %>%
         mutate(mean.bpm = mean(bpm, na.rm = TRUE)) %>%
         mutate(sd.bpm = sd(bpm, na.rm = TRUE)) %>%
         mutate(se.bpm = std.error(bpm, na.rm = TRUE)) %>%
         ungroup()

# CREATE GRAPH
bpm.graph <- ggplot(data = vf.df,
                    aes(x = order,
                        y = mean.bpm,
                        colour = turb.dis,
                        linetype = temp.dis,
                        shape = temp.dis)) +
              geom_point(size = 3,
                         position = position_dodge(width = 0.3)) +
              geom_line(position = position_dodge(width = 0.3)) +
              geom_point(aes(y = bpm),
                         alpha = 0.4,
                         position = position_jitterdodge(dodge.width = 0.5))+
              scale_colour_manual(labels = c("Clear", "Turbid"),
                                  values =c("darkturquoise","darkgoldenrod4"))+
              scale_linetype_manual(labels = c("16°C", "25°C"),
                                    values = c("solid", "dashed")) +
              geom_errorbar(aes(ymin = mean.bpm - se.bpm,
                                ymax = mean.bpm + se.bpm),
                            width = 0, position = position_dodge(width = 0.3))+
              labs(y = "Opercular beat rate (# opecular beat/min)",
                   colour = "Turbidity treatment",
                   linetype ="Thermal treatment",
                   title = NULL) +
              theme_classic()+
              theme(text = element_text(size = 18)) +
              scale_x_discrete(limits = c("Pre-ASR90", "Post-ASR90")) +
              theme(axis.title.x = element_blank()) +
              theme(legend.position = c(0.9, 0.2)) +
```

```
              theme(legend.text = element_text(size = 10)) +
              theme(axis.text.x = element_text(size = 18)) +
              theme(axis.text = element_text(size = 20)) +
              theme(legend.text = element_text(size=14)) +
              theme(legend.title = element_text(size=14)) +
              guides(shape = "none")

# CHECK ASSUMPTIONS FOR MODEL USING VF
# Normality
hist(vf.df$vf)
qqnorm(vf.df$vf)
qqline(vf.df$vf)

# Homogeneity of variance assumption
leveneTest(vf ~ treatment, data = vf.df)

table(vf.df[,"treatment"])
# Not equal sample size per treatment due to fish mortality

# CREATE MODEL
vf.glmm <- glmer(vf ~ pre.post + temp.dis*turb.dis + (pre.post | tank.number),
                 family = 'poisson',
                 data = vf.df)

summary(vf.glmm)
Anova(vf.glmm, type = 3)

#CHECK MODEL VALIDITY
# Dispersion
dispersion_glmer(vf.glmm)^2
# Dispersion parameter = 0.41, okay for Poisson distribution

# Homogeneity of variance
plot(resid(vf.glmm) ~ fitted(vf.glmm),
     xlab = "Predicted values",
     ylab = "Normalized residuals")
abline(h = 0, lty = 2)

# Normality
qqnorm(resid(vf.glmm))
hist(resid(vf.glmm))
```

# PUGNOSE SHINER CT_{max}, T_{ag}, AGITATION WINDOW, AND THERMAL SAFETY MARGIN ANALYSIS

```
# DOWNLOAD DATASET
ctmax.df <- read_csv2("DATA/ctmax.csv",
                      na = "empty",
                      trim_ws = TRUE)

# ADJUST DATA STRUCTURE
ctmax.df$temp.dis <- as.factor(ctmax.df$temp.dis)
ctmax.df$tank.number <- as.factor(ctmax.df$tank.number)
ctmax.df$turb.dis <- as.factor(ctmax.df$turb.dis)

# CREATE MEAN AND STANDARD ERROR DATA
ctmax.df <- ctmax.df %>%
            group_by(treatment) %>%
            mutate(mean.ctmax = mean(ctmax, na.rm = TRUE)) %>%
            mutate(se.ctmax = std.error(ctmax, na.rm = TRUE)) %>%
            mutate(sd.ctmax = sd(ctmax, na.rm = TRUE)) %>%
            mutate(mean.tag = mean(tag, na.rm = TRUE)) %>%
            mutate(se.tag = std.error(tag, na.rm = TRUE)) %>%
            mutate(sd.tag = sd(tag, na.rm = TRUE)) %>%
            mutate(mean.window = mean(window, na.rm = TRUE)) %>%
            mutate(se.window = std.error(window, na.rm = TRUE)) %>%
            mutate(sd.window = sd(window, na.rm = TRUE)) %>%
            mutate(mean.margin = mean(margin, na.rm = TRUE)) %>%
            mutate(se.margin = std.error(margin, na.rm = TRUE)) %>%
            mutate(sd.margin = sd(margin, na.rm = TRUE)) %>%
            ungroup()

# CREATE CTMAX GRAPH
ctmax.graph <- ggplot(data = ctmax.df,
                      aes(x = temp.dis,
                          y = mean.ctmax,
                          colour = turb.dis)) +
               geom_point(size = 3,
                          position = position_dodge(width = 0.3)) +
               geom_point(aes(y = ctmax),
                          alpha = 0.3,
                          size = 2,
                          position = position_jitterdodge(dodge.width = 0.3))+
               labs(x = NULL,
                    y = "Temperature (°C)",
                    title = bquote("A) CT"["max"]),
                    colour = " Turbidity treatment") +
               theme_classic() +
               geom_errorbar(aes(ymin = mean.ctmax - se.ctmax,
                                 ymax = mean.ctmax + se.ctmax),
                             width = 0,
                             position = position_dodge(width = 0.3)) +
               theme(plot.title = element_text(hjust = 0.5)) +
               scale_colour_manual(labels = c("Clear", "Turbid"),
                                   values=c("darkturquoise","darkgoldenrod4"))+
```

```
                theme(text = element_text(size = 20)) +
                scale_x_discrete(labels=c("16" = "16°C", "25" = "25°C")) +
                scale_y_continuous(breaks = c(30,32,34,36,38),
                                   limits = c(30,38)) +
                theme(axis.text = element_text(size = 20)) +
                theme(legend.position = c(0.25, 0.80)) +
                theme(legend.text = element_text(size = 13)) +
                theme(legend.title = element_text(size = 13))

tag.graph <- ggplot(data = ctmax.df,
                     aes(x = temp.dis,
                         y = mean.tag,
                         colour = turb.dis)) +
              geom_point(size = 3,
                         position = position_dodge(width = 0.3)) +
              geom_point(aes(y = tag),
                         alpha = 0.3,
                         size = 2,
                         position = position_jitterdodge(dodge.width = 0.3)) +
              labs(x = NULL,
                   y = NULL,
                   title = bquote("B) T"["ag"]),
                   colour = NULL) +
              theme_classic() +
              geom_errorbar(aes(ymin = mean.tag - se.tag,
                                ymax = mean.tag + se.tag),
                            width = 0,
                            position = position_dodge(width = 0.3)) +
              theme(plot.title = element_text(hjust = 0.5)) +
              scale_colour_manual(labels = c("Clear", "Turbid"),
                                  values = c("darkturquoise", "darkgoldenrod4"),
                                  guide = "none") +
              theme(text = element_text(size = 20)) +
              scale_x_discrete(labels = c("16" = "16°C", "25" = "25°C")) +
              theme(axis.text = element_text(size = 20)) +
              scale_y_continuous(breaks = seq(from = 26, to = 37, by = 2))

window.graph <- ggplot(data = ctmax.df,
                        aes(x = temp.dis,
                            y = mean.window,
                            colour = turb.dis)) +
                 geom_point(aes(y = window),
                            alpha = 0.3,
                            size = 2,
                            position = position_jitterdodge(dodge.width = 0.3))+
                 geom_point(size = 3,
                            position = position_dodge(width = 0.3)) +
                 labs(x = NULL,
                      y = "Temperature range (°C)",
                      title = "C) Agitation Window",
                      colour = NULL) +
                 theme_classic() +
                 geom_errorbar(aes(ymin = mean.window - se.window,
```

```
                          ymax = mean.window + se.window),
                      width = 0,
                      position = position_dodge(width = 0.3)) +
                theme(plot.title = element_text(hjust = 0.5)) +
                scale_colour_manual(labels = c("Clear", "Turbid"),
                                    values = c("darkturquoise",
                                               "darkgoldenrod4"),
                                    guide="none") +
                theme(text = element_text(size = 20)) +
                scale_x_discrete(labels = c("16" = "16°C", "25" = "25°C")) +
                theme(axis.text = element_text(size = 20)) +
                scale_y_continuous(breaks = seq(from = 0, to = 5, by = 1))

margin.graph <- ggplot(data = ctmax.df,
                       aes(x = temp.dis,
                           y = mean.margin,
                           colour = turb.dis)) +
                geom_point(size = 3,
                           position = position_dodge(width = 0.3)) +
                geom_point(aes(y = margin),
                           alpha = 0.3,
                           size = 2,
                           position = position_jitterdodge(dodge.width = 0.3))+
                labs(x = NULL,
                     y = NULL,
                     title = "D) Thermal Safety Margin",
                     colour = NULL) +
                theme_classic() +
                geom_errorbar(aes(ymin = mean.margin - se.margin,
                                  ymax = mean.margin + se.margin),
                              width = 0,
                              position = position_dodge(width = 0.3)) +
                theme(plot.title = element_text(hjust = 0.5)) +
                scale_colour_manual(labels = c("Clear", "Turbid"),
                                    values = c("darkturquoise",
                                               "darkgoldenrod4"),
                                    guide = "none") +
                theme(text = element_text(size = 20)) +
                scale_x_discrete(labels = c("16" = "16°C", "25" = "25°C")) +
                theme(axis.text = element_text(size = 20)) +
                scale_y_continuous(breaks = seq(from = 10, to = 16, by = 2))

combined.ctmax.graph <- ggarrange(ctmax.graph,
                                  tag.graph,
                                  window.graph,
                                  margin.graph,
                                  align = "v")

combined.ctmax.graph <- annotate_figure(combined.ctmax.graph,
                                   bottom = text_grob("Thermal treatment (°C)",
                                                      color = "black",
                                                      size = 20))
```

```
# CHECK ASSUMPTIONS FOR MODEL
# Normality assumption
qqnorm(ctmax.df$ctmax)
qqline(ctmax.df$ctmax)

qqnorm(ctmax.df$tag)
qqline(ctmax.df$tag)

qqnorm(ctmax.df$window)
qqline(ctmax.df$window)

qqnorm(ctmax.df$margin)
qqline(ctmax.df$margin)

# Homogeneity of variance assumption
leveneTest(ctmax ~ treatment, data = ctmax.df)
leveneTest(tag ~ treatment, data = ctmax.df)
leveneTest(window ~ treatment, data = ctmax.df)
leveneTest(margin ~ treatment, data = ctmax.df)
# Homogeneous variance

table(ctmax.df[,"treatment"])
# Not equal sample size due to fish mortality

# CHECK IF NEED RANDOM EFFECT IN MODEL
ctmax.lm <- lm(ctmax ~ temp.dis + turb.dis,
               data = ctmax.df)

avg.lm.test.resid <- rstandard(ctmax.lm)
plot(avg.lm.test.resid ~ as.factor(ctmax.df$tank.number),
     xlab = "tank number",
     ylab = "Standardized residuals")
abline(0, 0, lty = 2)
# Yes, this suggests a random effect of tank #

# CREATE MODELS
ctmax.lmer <- lmer(ctmax ~ temp.dis*turb.dis + ( 1 | tank.number),
                   data = ctmax.df)

summary(ctmax.lmer)
Anova(ctmax.lmer, type = 3)

tag.lmer <- lmer(tag ~ temp.dis*turb.dis + ( 1 | tank.number),
                 data = ctmax.df)

summary(tag.lmer)
Anova(tag.lmer, type = 3)

window.lmer <- lmer(window ~ temp.dis*turb.dis + ( 1 | tank.number),
                    data = ctmax.df)

summary(window.lmer)
```

```
Anova(window.lmer, type = 3)
margin.lmer <- lmer(margin ~ temp.dis*turb.dis + ( 1 | tank.number),
                    data = ctmax.df)

summary(margin.lmer)
Anova(margin.lmer, type = 3)

# CHECK MODEL VALIDATION

# Homogeneity of variance
plot(resid(ctmax.lmer) ~ fitted(ctmax.lmer),
     xlab = "Predicted values",
     ylab = "Normalized residuals")
abline(h = 0, lty = 2)

plot(resid(tag.lmer) ~ fitted(tag.lmer),
     xlab = "Predicted values",
     ylab = "Normalized residuals")
abline(h = 0, lty = 2)

plot(resid(window.lmer) ~ fitted(window.lmer),
     xlab = "Predicted values",
     ylab = "Normalized residuals")
abline(h = 0, lty = 2)

plot(resid(margin.lmer) ~ fitted(margin.lmer),
     xlab = "Predicted values",
     ylab = "Normalized residuals")
abline(h = 0, lty = 2)

# Normality
qqnorm(resid(ctmax.lmer))
hist(resid(ctmax.lmer))
densityPlot(resid(ctmax.lmer))

qqnorm(resid(tag.lmer))
hist(resid(tag.lmer))
densityPlot(resid(tag.lmer))

qqnorm(resid(window.lmer))
hist(resid(window.lmer))
densityPlot(resid(window.lmer))

qqnorm(resid(margin.lmer))
hist(resid(margin.lmer))
densityPlot(resid(margin.lmer))

# POST-HOC TESTS FOR MODELS WITH INTERACTIONS
emmeans(ctmax.lmer, list(pairwise ~ temp.dis*turb.dis), adjust = "holm")
summary(glht(ctmax.lmer, fct = mcp(temp.dis = "Tukey")), test =
adjusted("holm"))
```

APPENDIX II

# ENTEROMIUS NEUMAYERI CTM_{AX}/T_{AG}/WINDOW/TSM ANALYSIS

```
# DOWNLOAD DATASET
ctmax.df <- read_csv2("DATA/africa_ctmax.csv",
                      na = "empty",
                      trim_ws = TRUE)

# ADJUST DATA STRUCTURE
ctmax.df$pop <- as.factor(ctmax.df$pop)
ctmax.df$acc.time <- as.factor(ctmax.df$acc.time)
ctmax.df$tank <- as.factor(ctmax.df$tank)
ctmax.df$habitat <- as.factor(ctmax.df$habitat)

# REMOVE LONGTERM ACCLIMATION DATA
ctmax.df <- subset(ctmax.df, acc.time != "longterm")

# CREATE MEAN AND STANDARD ERROR DATA
ctmax.df <- ctmax.df %>%
            group_by(pop) %>%
            mutate(mean.ctmax = mean(ctmax, na.rm = TRUE)) %>%
            mutate(se.ctmax = std.error(ctmax, na.rm = TRUE)) %>%
            mutate(sd.ctmax = sd(ctmax, na.rm = TRUE)) %>%
            mutate(mean.tag = mean(tag, na.rm = TRUE)) %>%
            mutate(se.tag = std.error(tag, na.rm = TRUE)) %>%
            mutate(sd.tag = sd(tag, na.rm = TRUE)) %>%
            mutate(mean.window = mean(window, na.rm = TRUE)) %>%
            mutate(se.window = std.error(window, na.rm = TRUE)) %>%
            mutate(sd.window = sd(window, na.rm = TRUE)) %>%
            mutate(mean.margin = mean(margin, na.rm = TRUE)) %>%
            mutate(se.margin = std.error(margin, na.rm = TRUE)) %>%
            mutate(sd.margin = sd(margin, na.rm = TRUE)) %>%
            mutate(mean.max.margin = mean(max.margin, na.rm = TRUE)) %>%
            mutate(se.max.margin = std.error(max.margin, na.rm = TRUE)) %>%
            mutate(sd.max.margin = sd(max.margin, na.rm = TRUE)) %>%
            mutate(mean.weight = mean(weight, na.rm = TRUE)) %>%
            mutate(se.weight = std.error(weight, na.rm = TRUE)) %>%
            mutate(mean.std.length = mean(std.length, na.rm = TRUE)) %>%
            mutate(se.std.length = std.error(std.length, na.rm = TRUE)) %>%
            mutate(mean.total.length = mean(total.length, na.rm = TRUE)) %>%
            mutate(se.total.length = std.error(total.length, na.rm = TRUE)) %>%
            ungroup()

# CREATE CTMAX TRIAL GRAPH WITH SITE TEMPERATURE
ctmax.temp.graph <-ggplot(data = ctmax.df,
                          aes(x = site.temp,
                              y = mean.ctmax,
                              colour = habitat)) +
                    geom_point(aes(x = site.temp,
                                   y = ctmax),
                               alpha = 0.3,
                               position = position_dodge(width = 0.3)) +
```

```
                    geom_point(size = 2) +
                    labs(x = NULL,
                         y = "Temperature (°C)",
                         title = bquote("A) CT"["max"]),
                         colour ="Habitat type") +
                    theme_classic() +
                    geom_errorbar(aes(ymin = mean.ctmax - se.ctmax,
                                      ymax = mean.ctmax + se.ctmax),
                                  width = 0,
                                  position = position_dodge(width = 0.3)) +
                    theme(plot.title = element_text(hjust = 0.5)) +
                    theme(legend.position = "bottom") +
                    theme(text = element_text(size = 20))+
                    scale_colour_manual(labels = c("Stream", "Swamp"),
                                        values = c("darkorange","darkorchid"))+
                    scale_x_continuous(breaks = c(17.8, 18.4, 19)) +
                    theme(legend.position = "none")

tag.temp.graph <-ggplot(data = ctmax.df,
                        aes(x = site.temp,
                            y = mean.tag,
                            colour = habitat)) +
                geom_point(aes(x = site.temp,
                               y = tag),
                           alpha = 0.3,
                           position = position_dodge(width = 0.3)) +
                geom_point(size = 2) +
                labs(x = NULL,
                     y = NULL,
                     title = bquote("B) T"["ag"]),
                     colour = "Habitat type") +
                theme_classic() +
                geom_errorbar(aes(ymin = mean.tag - se.tag,
                                  ymax = mean.tag + se.tag),
                              width = 0,
                              position = position_dodge(width = 0.3)) +
                theme(plot.title = element_text(hjust = 0.5)) +
                theme(text = element_text(size = 20))+
                scale_colour_manual(labels = c("Stream", "Swamp"),
                                    values = c("darkorange", "darkorchid")) +
                scale_x_continuous(breaks = c(17.8, 18.4, 19)) +
                theme(legend.position = "none")

window.temp.graph <-ggplot(data = ctmax.df,
                           aes(x = site.temp,
                               y = mean.window,
                               colour = habitat)) +
                    geom_point(aes(x = site.temp,
                                   y = window),
                               alpha = 0.3,
                               position = position_dodge(width = 0.3)) +
                    geom_point(size = 2) +
                    labs(x = NULL,
```

```
                        y = "Temperature range (°C)",
                        title = bquote("C) Agitation Window"),
                        colour = "Habitat type") +
                   theme_classic() +
                   geom_errorbar(aes(ymin = mean.window - se.window,
                                     ymax = mean.window + se.window),
                                 width = 0,
                                 position = position_dodge(width = 0.3)) +
                   theme(plot.title = element_text(hjust = 0.5)) +
                   theme(text = element_text(size = 20))+
                   scale_colour_manual(labels = c("Stream", "Swamp"),
                                       values = c("darkorange","darkorchid"))+
                   scale_x_continuous(breaks = c(17.8,18.4,19)) +
                   theme(legend.position = "none")

margin.temp.graph <-ggplot(data = ctmax.df,
                          aes(x = site.temp,
                              y = mean.max.margin,
                              colour = habitat)) +
                   geom_point(aes(x = site.temp,
                                  y = max.margin),
                              alpha = 0.3,
                              position = position_dodge(width = 0.3)) +
                   geom_point(size = 2) +
                   labs(x = NULL,
                        y = NULL,
                        title = bquote("D) Thermal safety margin"),
                        colour ="Habitat type") +
                   theme_classic() +
                   geom_errorbar(aes(ymin = mean.max.margin - se.max.margin,
                                     ymax = mean.max.margin + se.max.margin),
                                 width = 0,
                                 position = position_dodge(width = 0.3)) +
                   theme(plot.title = element_text(hjust = 0.5)) +
                   theme(text = element_text(size = 20))+
                   scale_colour_manual(labels = c("Stream / River ", "Swamp"),
                                       values = c("darkorange","darkorchid"))+
                   scale_x_continuous(breaks = c(17.8,18.4,19)) +
                   theme(legend.position = c(0.26, 0.16)) +
                   theme(legend.text = element_text(size = 14)) +
                   theme(legend.title = element_text(size = 14))

combined.ctmax.temp.graph <- ggarrange(ctmax.temp.graph,
                                       tag.temp.graph,
                                       window.temp.graph,
                                       margin.temp.graph,
                                       align = "v")

combined.ctmax.temp.graph <- annotate_figure(combined.ctmax.temp.graph,
                                             bottom = text_grob("Average site
                                                           temperature (°C)",
                                                           color = "black",
                                                           size = 20))
```

```
# CREATE CTMAX TRIAL GRAPH WITH SITE DO
ctmax.do.graph <-ggplot(data = ctmax.df,
                        aes(x = site.do,
                            y = mean.ctmax,
                            colour = habitat)) +
                  geom_point(aes(x = site.do,
                                 y = ctmax),
                             alpha = 0.3,
                             position = position_dodge(width = 0.3)) +
                  geom_point(size = 2) +
                  labs(x = NULL,
                       y = "Temperature (°C)",
                       title = bquote("A) CT"["max"]),
                       colour = "Habitat type") +
                  theme_classic() +
                  geom_errorbar(aes(ymin = mean.ctmax - se.ctmax,
                                    ymax = mean.ctmax + se.ctmax),
                                width = 0, position = position_dodge(width =
                                       0.3)) +
                  theme(plot.title = element_text(hjust = 0.5)) +
                  theme(legend.position = "bottom") +
                  theme(text = element_text(size = 20))+
                  scale_colour_manual(labels = c("Stream / River", "Swamp"),
                                      values = c("darkorange", "darkorchid")) +
                  theme(legend.position = "none")

tag.do.graph <-ggplot(data = ctmax.df,
                      aes(x = site.do,
                          y = mean.tag,
                          colour = habitat)) +
                  geom_point(aes(x = site.do,
                                 y = tag),
                             alpha = 0.3,
                             position = position_dodge(width = 0.3)) +
                  geom_point(size = 2) +
                  labs(x = NULL,
                       y = NULL,
                       title = bquote("B) T"["ag"]),
                       colour = "Habitat type") +
                  theme_classic() +
                  geom_errorbar(aes(ymin = mean.tag - se.tag,
                                    ymax = mean.tag + se.tag),
                                width = 0,
                                position = position_dodge(width = 0.3)) +
                  theme(plot.title = element_text(hjust = 0.5)) +
                  theme(text = element_text(size = 20))+
                  scale_colour_manual(labels = c("Stream / River", "Swamp"),
                                      values = c("darkorange", "darkorchid")) +
                  theme(legend.position = c(0.80, 0.15)) +
                  theme(legend.text = element_text(size = 13)) +
                  theme(legend.title = element_text(size = 13))
```

```
window.do.graph <-ggplot(data = ctmax.df,
                         aes(x = site.do,
                             y = mean.window,
                             colour = habitat)) +
                  geom_point(aes(x = site.do,
                                 y = window),
                             alpha = 0.3,
                             position = position_dodge(width = 0.3)) +
                  geom_point(size = 2) +
                  labs(x = NULL,
                       y = "Temperature range (°C)",
                       title = bquote("C) Agitation Window"),
                       colour ="Habitat type") +
                  theme(legend.position = "bottom" ) +
                  theme_classic() +
                  geom_errorbar(aes(ymin = mean.window - se.window,
                                    ymax = mean.window + se.window),
                                width = 0,
                                position = position_dodge(width = 0.3)) +
                  theme(plot.title = element_text(hjust = 0.5)) +
                  theme(legend.position = "bottom") +
                  theme(text = element_text(size = 20))+
                  scale_colour_manual(labels = c("Stream", "Swamp"),
                                      values = c("darkorange", "darkorchid")) +
                  theme(legend.position = "none")

margin.do.graph <-ggplot(data = ctmax.df,
                         aes(x = site.do,
                             y = mean.max.margin,
                             colour = habitat)) +
                    geom_point(aes(x = site.do,
                                   y = max.margin),
                               alpha = 0.3,
                               position = position_dodge(width = 0.5)) +
                    geom_point(size = 2) +
                    labs(x = NULL,
                         y = NULL,
                         title = bquote("D) Thermal safety margin"),
                         colour = "Habitat type") +
                    theme_classic() +
                    geom_errorbar(aes(ymin = mean.max.margin - se.max.margin,
                                      ymax = mean.max.margin + se.max.margin),
                                  width = 0,
                                  position = position_dodge(width = 0.3)) +
                    theme(plot.title = element_text(hjust = 0.5)) +
                    theme(text = element_text(size = 20)) +
                    scale_colour_manual(labels = c("Stream", "Swamp"),
                                        values = c("darkorange","darkorchid"))+
                    theme(legend.position = "none")
```

```
combined.ctmax.do.graph <- ggarrange(ctmax.do.graph,
                                     tag.do.graph,
                                     window.do.graph,
                                     margin.do.graph,
                                     align = "v")

combined.ctmax.do.graph <- annotate_figure(combined.ctmax.do.graph,
                                          bottom = text_grob("Average site
                                                     dissolved oxygen (mg/l)",
                                                              color = "black",
                                                              size = 20))

# CHECK ASSUMPTIONS FOR MODEL
# Normality assumption
qqnorm(ctmax.df$ctmax)
qqline(ctmax.df$ctmax)

qqnorm(ctmax.df$tag)
qqline(ctmax.df$tag)

qqnorm(ctmax.df$window)
qqline(ctmax.df$window)

qqnorm(ctmax.df$max.margin)
qqline(ctmax.df$max.margin)
# All parameters are normally distributed

table(ctmax.df[,"pop"])
# Samples are not equal in size due to fish mortality

# Homogeneity of variance assumption
leveneTest(ctmax ~ pop, data = ctmax.df)
leveneTest(tag ~ pop, data = ctmax.df)
leveneTest(window ~ pop, data = ctmax.df)
leveneTest(max.margin ~ pop, data = ctmax.df)
# All parameters have homogeneous variance

# Check if population/site random effect is needed
ctmax.lm <- lm(ctmax ~ pop,
               data = ctmax.df)

avg.lm.test.resid <- rstandard(ctmax.lm)

plot(avg.lm.test.resid ~ as.factor(ctmax.df$pop),
     xlab = "population",
     ylab = "Standardized residuals")
abline(0, 0, lty = 2)
# Population/site seems to have a small effect on CTmax so models should
include random population effect

# CREATE MODELS
ctmax.lmer <- lmer(ctmax ~ site.temp + site.do + weight + ( 1 | pop),
                   data = ctmax.df)
```

```
summary(ctmax.lmer)
Anova(ctmax.lmer, type = "III")
#Type 3 because of unequal sample size

tag.lmer <- lmer(tag ~ site.temp + site.do + weight + ( 1 | pop),
                   data = ctmax.df)

summary(tag.lmer)
Anova(tag.lmer, type = 'III')

window.lmer <- lmer(window ~ site.temp + site.do + weight + ( 1 | pop),
                 data = ctmax.df)

summary(window.lmer)
Anova(window.lmer, type = 'III')

max.margin.lmer <- lmer(max.margin ~ site.temp + site.do + weight + ( 1 | pop),
                    data = ctmax.df)

summary(max.margin.lmer)
Anova(max.margin.lmer, type = 'III')

# CHECK MODEL VALIDATION
# Homogeneity of variance
plot(resid(ctmax.lmer) ~ fitted(ctmax.lmer),
     xlab = "Predicted values",
     ylab = "Normalized residuals")
abline(h = 0, lty = 2)

plot(resid(tag.lmer) ~ fitted(tag.lmer),
     xlab = "Predicted values",
     ylab = "Normalized residuals")
abline(h = 0, lty = 2)

plot(resid(window.lmer) ~ fitted(window.lmer),
     xlab = "Predicted values",
     ylab = "Normalized residuals")
abline(h = 0, lty = 2)

plot(resid(margin.lmer) ~ fitted(margin.lmer),
     xlab = "Predicted values",
     ylab = "Normalized residuals")
abline(h = 0, lty = 2)

# Normality
qqnorm(resid(ctmax.lmer))
hist(resid(ctmax.lmer))
densityPlot(resid(ctmax.lmer))

qqnorm(resid(tag.lmer))
hist(resid(tag.lmer))
densityPlot(resid(tag.lmer))
```

```
qqnorm(resid(window.lmer))
hist(resid(window.lmer))
densityPlot(resid(window.lmer))

qqnorm(resid(margin.lmer))
hist(resid(margin.lmer))
densityPlot(resid(margin.lmer))
```
